# Regional variations in childbirth interventions in the Netherlands: a nationwide explorative study

**DOI:** 10.1186/s12884-018-1795-0

**Published:** 2018-06-01

**Authors:** A. E. Seijmonsbergen-Schermers, D. C. Zondag, M. Nieuwenhuijze, T. Van den Akker, C. J. Verhoeven, C. Geerts, F. Schellevis, A. De Jonge

**Affiliations:** 1 Department of Midwifery Science, AVAG, Amsterdam Public Health research Institute, VU University Medical Center, Van der Boechorststraat 7, 1081 BT Amsterdam, the Netherlands; 2 Research Centre for Midwifery Science, Zuyd University, Universiteitssingel 60, 6229 ER Maastricht, the Netherlands; 3 Department of Obstetrics, Leiden University Medical Center, Albinusdreef 2, 2333 ZA Leiden, the Netherlands; 4 Department of Obstetrics and Gynaecology, Maxima Medical Centre, De Run 4600, PO Box 7777, 5500 MB Veldhoven, the Netherlands; 5 NIVEL (Netherlands Institute for Health Services Research), PO Box 1568, 3500 BN Utrecht, the Netherlands; 6 Department of General Practice & Elderly Care Medicine, Amsterdam Public Health Research Institute, VU University Medical Center, Amsterdam, the Netherlands

**Keywords:** Childbirth, Interventions, Obstetric, Regional, Variations, Outcomes, Caesarean section, Induction, Pain relief

## Abstract

**Background:**

Although interventions in childbirth are important in order to prevent neonatal and maternal morbidity and mortality, non-indicated use may cause avoidable harm. Regional variations in intervention rates, which cannot be explained by maternal characteristics, may indicate over- and underuse. The aim of this study is to explore regional variations in childbirth interventions in the Netherlands and their associations with interventions and adverse outcomes, controlled for maternal characteristics.

**Methods:**

Childbirth intervention rates were compared between twelve Dutch regions, using data from the national perinatal birth register for 2010–2013. All single childbirths from 37 weeks’ gestation onwards were included. Primary outcomes were induction and augmentation of labour, pain medication, instrumental birth, caesarean section (prelabour, intrapartum) and paediatric involvement. Secondary outcomes were adverse neonatal and maternal outcomes. Multivariable logistic regression analyses were used to adjust for maternal characteristics. Associations were expressed in Spearman’s rank correlation coefficients.

**Results:**

Most variation was found for type of pain medication and paediatric involvement. Epidural analgesia rates varied from between 12 and 38% (nulliparous) and from between 5 and 14% (multiparous women). These rates were negatively correlated with rates of other pharmacological pain relief, which varied from between 15 and 43% (nulliparous) and from between 10 and 27% (multiparous). Rates of paediatric involvement varied from between 37 and 60% (nulliparous) and from between 26 and 43% (multiparous). For instrumental vaginal births, rates varied from between 16 and 19% (nulliparous) and from between 3 and 4% (multiparous). For intrapartum caesarean section, the variation was 13–15% and 5–6%, respectively. A positive correlation was found between intervention rates in midwife-led and obstetrician-led care at the onset of labour within the same region. Adverse neonatal and maternal outcomes were not lower in regions with higher intervention rates. Higher augmentation of labour rates correlated with higher rates of severe postpartum haemorrhage.

**Conclusions:**

Most variation was found for type of pain medication and paediatric involvement, and least for instrumental vaginal births and intrapartum caesarean sections. Care providers and policy makers should critically audit remarkable variations, since these may be unwarranted. Limited variation for some interventions may indicate consensus for their use. Further research should focus on variations in evidence-based interventions and indications for the use of interventions in childbirth.

**Electronic supplementary material:**

The online version of this article (10.1186/s12884-018-1795-0) contains supplementary material, which is available to authorized users.

## Background

The rates of interventions in childbirth vary worldwide [1–4] and have fluctuated over the years [1, 4–7]. Induction of labour and caesarean section (CS) rates have shown a steady increase since the 1970s [1, 4, 6, 8, 9], which raised concerns [10]. Interventions in childbirth are important in order to prevent neonatal and maternal morbidity and mortality. However, use without a medical indication may cause avoidable harm [2, 11–14]. The World Health Organization (WHO) recommends limited use of interventions during childbirth [15]. Induction and augmentation of labour should only be performed on medical indication [16, 17]. However, there are concerns about poor adherence to this recommendation in a significant number of women with uncomplicated pregnancies [16–19]. Epidural analgesia is the most effective method for pain medication during labour [20], but is associated with a higher risk of instrumental birth, oxytocin use, maternal fever, urinary retention and complications, such as post-dural puncture headache [20, 21]. The decision for pain medication is ultimately based on women’s choice. There is some evidence that continuous support of labour might reduce the need for pain medication [22]. Furthermore, the WHO states that CS rates higher than 10 % at population level are not associated with reductions in maternal, neonatal and infant mortality rates [23].

Variations in intervention rates between high-income countries may be explained by culture and history, differences in population characteristics, maternity care systems, and national guidelines [12, 15, 24–26]. Clinical guidelines have been used for a long time to harmonise and rationalise the use of interventions within countries, and to improve outcomes [27, 28]. Nevertheless, studies comparing regions within countries like England, Ireland, Canada and Germany, have found substantial variations in rates of induction of labour, epidural analgesia, continuous fetal electronic monitoring, episiotomy, instrumental birth, and CS [29–33]. Additionally, Dutch studies have reported variations in rates between hospitals, of induction and augmentation of labour, administration of sedation and analgesics, episiotomy, instrumental birth, and CS [34, 35]. Regional variations in intervention rates, which cannot be explained by maternal characteristics, may indicate over- and underuse [36]. This is especially true in a relatively small country without regional differences in the maternity healthcare system.

The aim of this study was therefore to explore which regional variations in intervention rates in childbirth exist, and how these variations are associated both to each other, and to adverse neonatal and maternal outcomes. These are explored for single childbirths from 37 weeks of gestation onwards in midwife- or obstetrician-led care in the Netherlands, and controlled for maternal characteristics.

## Methods

### Data collection

For this nationwide study, we used consolidated data of the years 2010 to 2013 from Perined, the national perinatal register that includes data from almost all births in the Netherlands. Perined aims to improve the quality of perinatal care through providing data for research and audits on adverse outcomes. The Perined register includes data from: primary midwife-led care (the national perinatal database 1); secondary obstetrician-led care (the national perinatal database 2); paediatric care (the national neonatal register); and primary midwifery care by general practitioners (the national perinatal database h). The data are routinely recorded by the care providers and combined into the Perined register via a validated linkage method [37, 38]. More than 98% of all midwifery practices and obstetric hospital units record their births in this combined database [39]. All single childbirths from 37 weeks’ gestation onwards were included. Exclusion criteria were missing data on: postal code; parity; or from the national perinatal database 1, covering midwife-led care, but where the woman was referred to obstetrician-led care, covered by the national database 2.

In the Netherlands, low-risk women in primary midwife-led care are cared for by independent midwives who attend home births, low-risk hospital births, and births in alongside and free-standing birth centres. The Dutch Birth Centre Study showed that health outcomes, experiences, and costs for low-risk women are similar for planned birth in a birth centre and planned birth in a hospital, both supervised by a primary care midwife [40, 41]. When risks for adverse outcomes increase or complications arise, women are referred to obstetrician-led care. Interventions in childbirth such as induction and augmentation of labour, pain medication, instrumental birth, and CS, are only available in an obstetrician-led care setting [42, 43]. Intrapartum interventions may be used for women in midwife-led care at the onset of labour after referral to obstetrician-led care. Therefore, intervention rates are not comparable for women who are in midwife-led care and women who are in obstetrician-led care at the onset of labour.

The VU University Medical Center confirmed that ethical approval was not required for this study according to the Dutch legislation (reference WC2016–055; http://www.ccmo.nl/en/your-research-does-it-fall-under-the-wmo).

### Interventions

Births were attributed to one of the twelve Dutch administrative provinces (further referred to as ‘regions’) according to the residential postal code of the mother. All low-risk women have access to all types of birth settings, but not all types are present in all regions [44]. We adjusted for this by using the residential postal code of the mother.

The following interventions were examined as the primary outcomes: induction of labour; augmentation after a spontaneous onset of labour; intrapartum oxytocin use; epidural analgesia; other pharmacological pain relief; instrumental vaginal birth; CS (prelabour, intrapartum); and involvement of a paediatrician in the first 24 h after birth. Births from 42 weeks onwards were not excluded, because they may explain variation in particularly induction of labour rates, and they may reflect different policies between regions. Artificial rupture of membranes before a spontaneous onset of labour was defined as induction of labour, and administration of oxytocin to stimulate uterine contractions after spontaneously ruptured membranes as augmentation. A CS after spontaneously ruptured membranes was defined as intrapartum CS. Intrapartum oxytocin includes the use of oxytocin for induction or for augmentation of labour, but not oxytocin use in the third stage of labour. Women with a prelabour CS were excluded from the analyses on pain medication. Women with an intrapartum CS and an epidural, are classified as epidural analgesia for labour pain, since epidural analgesia is generally not used for caesarean sections without prior epidural analgesia for labour pain. In Perined ‘other pharmacological pain relief’ is specified as: sedatives; non-opioid analgesics; and opioid analgesics without further details. The most common opioid analgesics are pethidine injections, sometimes combined with a sedative such as promethazine, and patient-controlled remifentanil [45]. In some births, epidural analgesia and other pharmacological methods for pain medication were both used, and therefore, the percentages could not be added up [45].

### Neonatal and maternal outcomes

The secondary neonatal and maternal outcomes were: antepartum and intrapartum stillbirth; neonatal mortality; Apgar score below 7 at 5 min; third or fourth degree perineal tear among vaginal births; and postpartum haemorrhage (PPH) of 1000 ml or more. Antepartum stillbirths with births beyond 37 weeks were included, since this may influence intervention rates. Neonatal mortality was defined as neonatal death up to 7 days. Antepartum and intrapartum stillbirths were excluded from the analyses on Apgar score. Women who gave birth by CS were excluded from the analyses on third or fourth degree perineal tear.

### Maternal and neonatal characteristics

The following maternal and neonatal characteristics were included as independent variables or potential confounders [29, 30, 32, 46–49]: parity (nulliparous, multiparous); care setting at the onset of labour (midwife-led, obstetrician-led), maternal age (< 20, 20–24, 25–29, 30–34, 35–39, ≥40 years); ethnic background (Dutch, non-Dutch); degree of urbanisation (urban, intermediate, rural); socioeconomic status (high, medium, low); gestational age (37 + 0–37 + 6, 38 + 0–40 + 6, 41 + 0–41 + 6, ≥42 weeks); and birth weight (< 2.3rd, <10th, >90th, > 97.7th percentile). Ethnic background was reported by the care provider and was defined as Dutch or non-Dutch, because of inconsistencies in recording non-Dutch subgroups. The degree of urbanisation was based on the four digits of the residential postal code of the mother. For 2500 or more addresses/km^2^, the degree of urbanisation was categorized as urban, and for less than 500 addresses/km^2^ as rural. Socioeconomic status [SES] was based on a proxy measure indicated by the Netherlands Institute for Social Research (SCP), which includes education, employment, and level of income of the residential postal code area (Statistics Netherlands; https://bronnen.zorggegevens.nl/Bron?naam=Sociaal-Economische-Status-per-postcodegebied). SES was classified as high, medium and low, based on the 25 and 75 percentile cut-off points.

### Data analysis

The baseline characteristics were described in percentages per region. The variation in interventions was analysed overall, and in subgroups according to the care setting. Stratification by parity was applied for the crude rates. Univariable analyses were performed to gain insight in the variations of intervention rates and childbirth outcomes in the twelve regions. All interventions and childbirth outcomes mentioned above were included in the univariable analyses. The percentages of missing data were low, namely from between 0.0 to 2.5% for baseline characteristics, from between 0.0 to 0.8% for interventions, from between 0.0 to 0.1% for neonatal outcomes, and from between 1.4 to 2.7% for maternal outcomes. Therefore, cases with missing data were excluded.

Multivariable logistic regression analyses were conducted for all births and stratified by the care setting, with adjustments for: parity; maternal age; ethnic background; socioeconomic position; and the degree of urbanisation. The results of the multivariable analyses were illustrated in figures with maps and boxplots with adjusted odds ratios (ORs) and 99% confidence intervals (CIs). The weighted overall intervention rate was taken as the reference. This weighted rate was the overall intervention rate, with the intervention rate of the region weighted for the number of women in each region. A confidence interval of 99% was chosen to limits chance findings due to multiple testing in a large dataset. Outcome variables were dichotomised and dummy variables were created to account for potential confounders in the multivariable logistic regression analyses. An important topic of this study, was to explore whether the variation of one intervention was associated with the variation of another intervention. Instead of exploring associations with eyeballing only, we quantified these associations by calculating Spearman’s rank correlation coefficients. These were calculated to demonstrate the associations of regional adjusted ORs between interventions in different care settings, and between interventions and childbirth outcomes. Correlation coefficients were calculated for the adjusted ORs of the regions, but only for outcomes that varied significantly between the regions. Since the sample size for all calculated correlations was the same, namely 12 regions, all correlations with ρ ≥ 0.57 or ≤ − 0.57 corresponded with a *p*-value of 0.05. Although the limits for clinically significant correlations are arbitrary, we considered a correlation of ρ ≥ 0.60 or ≤ − 0.60 as strong [50], and only these correlations were discussed in the text and indicated in bold in the tables.

Statistical analyses were performed using SPSS Statistics 22 (SPSS Inc., Chicago, IL, USA).

First, overall results and remarkable associations between subgroups of women or between interventions were described. Second, results for each intervention were described, starting with those that showed most variation.

## Results

### Baseline characteristics

**Fig. 1:**
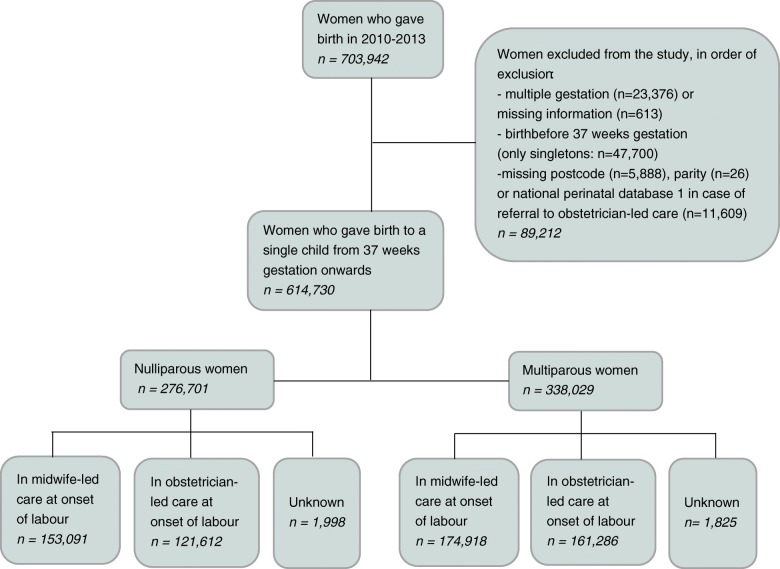
Study population

Figure 1 shows the number of births eligible for inclusion in this study and Table 1 describes the maternal and neonatal characteristics. Of the 276,701 births in nulliparous women, 153,091 were in midwife-led care at the onset of labour, 121,612 in obstetrician-led care, and for the remainder, the care setting was unknown. For births in multiparous women, these numbers were 174,918 and 161,286 respectively. In the regions, the proportion of mothers younger than 20 years of age ranged from between 0.8 to 2.2%, and of 40 years or older from between 2.4 to 4.5%. The lowest proportion of mothers with a non-Dutch ethnicity was 9.3% and the highest 34.6%. In three regions, there were no urban areas, whereas in all regions there were mothers living in rural areas, with a range of between 11.1 and 48.5%. Proportions of mothers with a low socioeconomic status varied from between 24.1 to 59.2%. Regions with the lowest number of births after 42 weeks (varying from between 0.8 to 2.5%), had higher numbers of births at 37–38 weeks (varying from between 5.8 to 9.2%), and vice versa. We found a similar pattern for birth weight below the 2.3rd, 10th or above the 90th or 97.7th percentile, with rates varying from between 1.4 to 2.1% for birth weight below the 2.3rd percentile, and from between 2.3 to 3.5% for birth weight above the 97.7th.

**Table 1:** Maternal and neonatal characteristics of women by region

	GR	FR	DR	OV	FL	GD	UT	NH	ZH	ZL	NB	LB
Total *n*	19,441	22,568	15,875	42,869	17,461	71,286	52,893	105,948	139,573	11,327	84,187	31,302
Parity, *%*												
Nulliparous	45.8	42.4	42.7	42.1	41.4	43.4	44.7	46.8	45.7	42.8	45.8	47.2
Multiparous	54.2	57.6	57.3	57.9	58.6	56.6	55.3	53.2	54.3	57.2	54.2	52.8
Maternal age, %												
< 20 years	1.9	1.5	1.6	1.2	2.2	1.2	0.8	1.0	1.5	1.6	1.0	1.6
20–24 years	12.4	11.5	11.6	10.0	13.9	10.0	7.5	8.4	11.7	15.2	8.7	10.6
25–29 years	31.2	34.8	35.4	33.4	33.9	31.6	26.7	26.2	30.3	33.9	31.5	32.2
30–34 years	35.4	35.1	34.2	38.0	32.3	37.5	40.6	38.2	35.6	33.1	39.8	37.8
35–39 years	16.1	14.5	14.7	14.9	14.6	16.6	20.9	21.6	17.4	13.6	16.5	15.2
≥40 years	2.9	2.7	2.5	2.4	3.0	3.1	3.6	4.5	3.5	2.7	2.5	2.7
Ethnic background, %												
Dutch	85.7	90.7	89.9	86.2	65.4	85.6	77.5	67.1	65.4	87.2	80.1	82.7
Non-Dutch	14.3	9.3	10.1	13.8	34.6	14.4	22.5	32.9	34.6	12.8	19.9	17.3
Urbanisation, *%*												
Urban	18.0	4.6	0.0	2.9	0.0	3.8	23.0	39.8	41.7	0.0	9.0	2.5
Intermediate	49.2	47.0	53.6	71.3	72.5	71.4	59.6	49.1	45.0	53.3	70.0	69.7
Rural	32.9	48.5	46.4	25.9	27.5	24.8	17.4	11.1	13.3	46.7	21.0	27.8
Socioeconomic status, *%*												
High (*p* ≥ 75)	9.3	11.8	19.4	16.9	39.2	19.1	35.7	23.5	25.5	7.4	20.0	8.7
Medium (p 25–75)	31.5	34.8	40.5	51.4	36.8	56.0	38.8	39.0	39.1	60.3	55.8	58.4
Low (*p* ≤ 25)	59.2	53.4	40.0	31.7	24.1	24.9	25.4	37.5	35.4	32.2	24.1	32.9
Gestational age (weeks), *%*												
37 + 0–37 + 6	8.7	8.3	9.2	8.6	8.4	6.7	5.8	6.6	7.6	6.5	7.3	8.8
38 + 0–40 + 6	71.5	71.7	72.5	72.1	73.1	71.6	71.2	72.2	72.8	71.6	72.4	73.9
41 + 0–41 + 6	17.9	18.1	16.9	17.4	16.9	19.3	20.7	19.0	18.3	19.4	18.5	16.5
≥42	1.8	1.9	1.4	1.8	1.6	2.3	2.3	2.2	1.4	2.5	1.8	0.8
Birth weight, *%*												
< 2,3rd percentile	1.7	1.4	1.4	1.5	1.8	1.6	1.7	1.8	1.9	2.0	2.0	2.1
<10th percentile	8.0	6.8	7.3	7.4	9.5	7.8	7.9	8.4	8.9	8.8	9.3	9.7
>90th percentile	11.3	12.9	11.9	11.0	9.8	11.2	10.6	10.3	9.7	10.0	9.0	9.0
> 97,7th percentile	3.0	3.5	3.4	2.9	2.4	3.0	2.7	2.7	2.5	2.4	2.3	2.4

Percentage of missing data: 0.0% for maternal age, 0.4% for ethnic background, 1.1% for urbanisation, 2.5% for socioeconomic status, 0.2% for birth weight

### Results on the national level

**Fig. 2:**
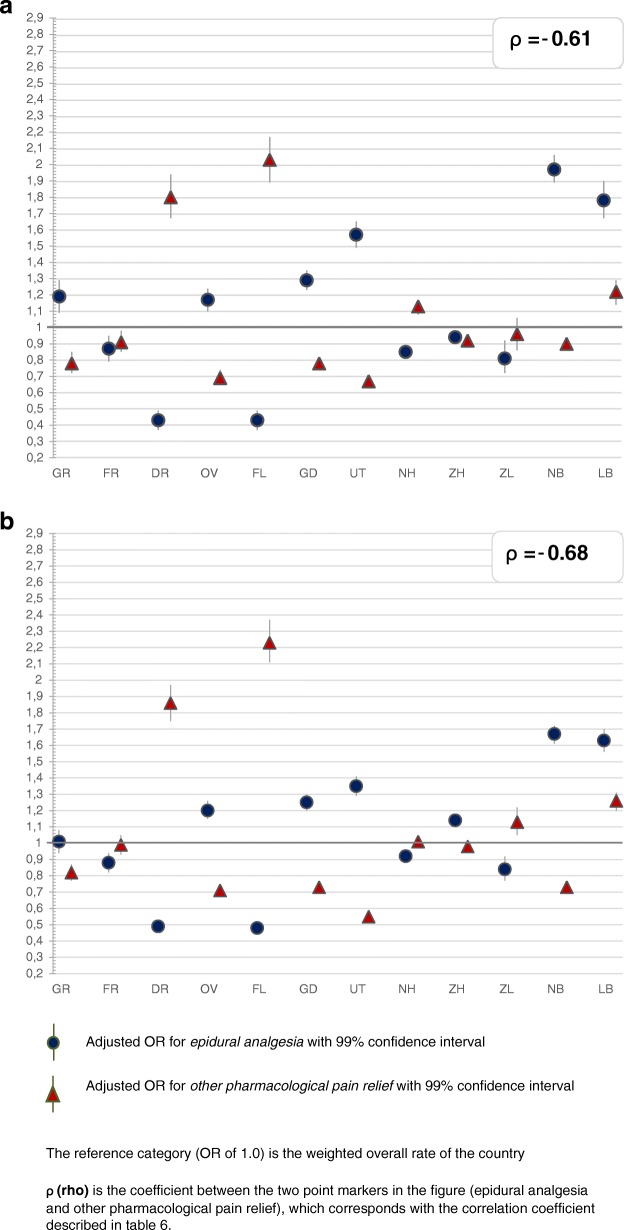
**a**: Regional variation of epidural analgesia and other pharmacological pain relief for women in midwife-led care. **b**: Regional variation of epidural analgesia and other pharmacological pain relief for women in obstetrician-led care

**Fig. 3:**
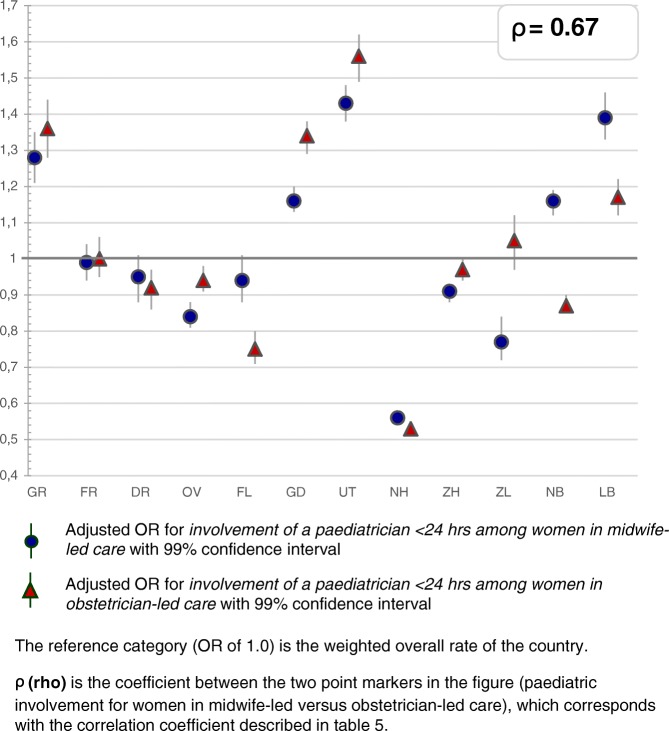
Regional variation of paediatric involvement for women in midwife-led care and obstetrician-led care

**Fig. 4:**
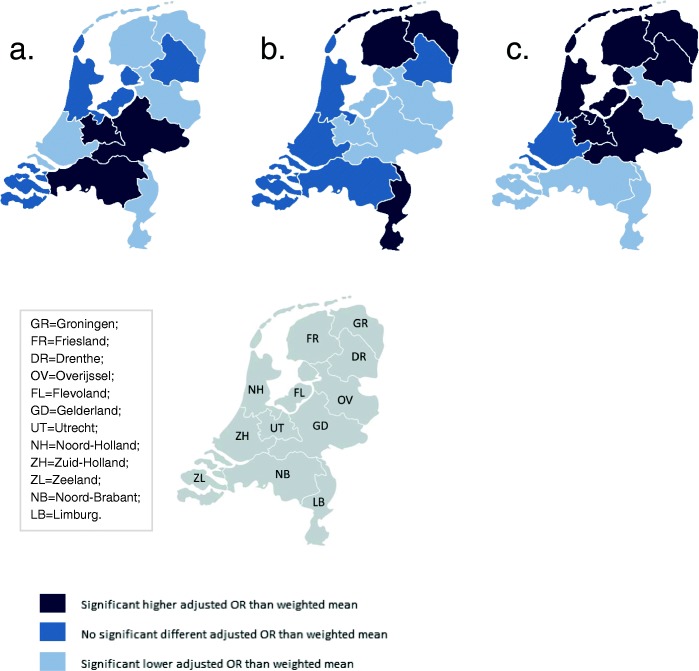
Significant differences in adjusted* OR between regions in incidences of: **a**. spontaneous births. **b**. caesarean sections. **c**. intrapartum oxytocin use. (* Adjusted for parity, maternal age, ethnic background, socioeconomic status and urbanisation)

**Fig. 5:**
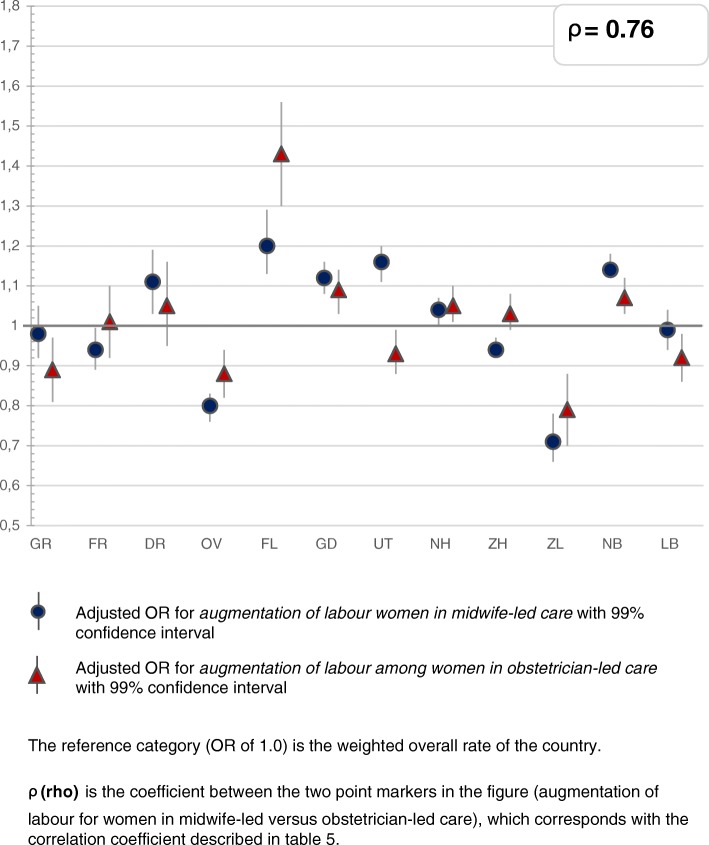
Regional variation of augmentation of labour after spontaneous onset for women in midwife-led care and obstetrician-led care

**Fig. 6:**
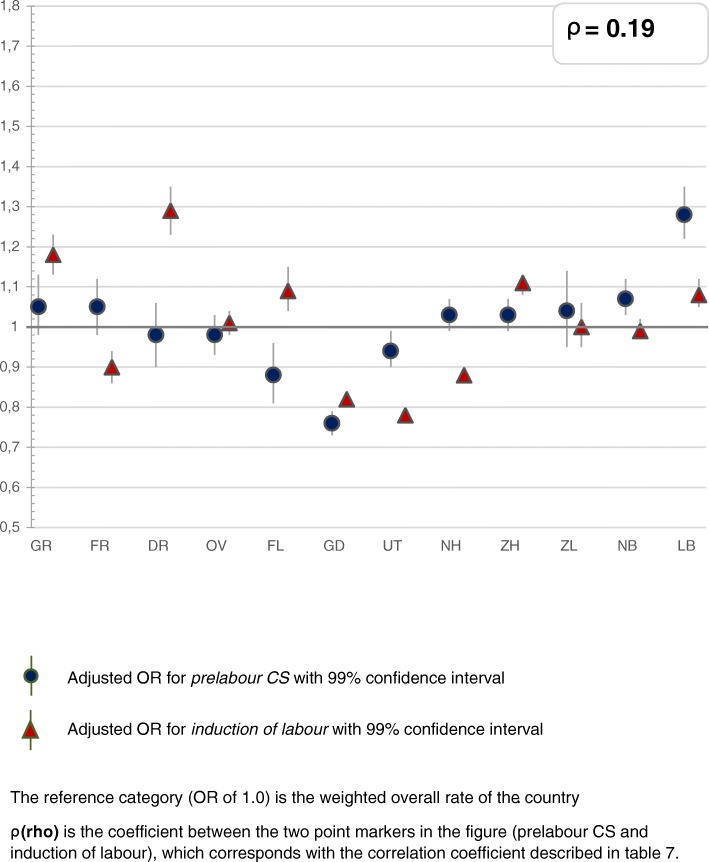
Regional variation of prelabour CS and induction of labour for all women

**Fig. 7:**
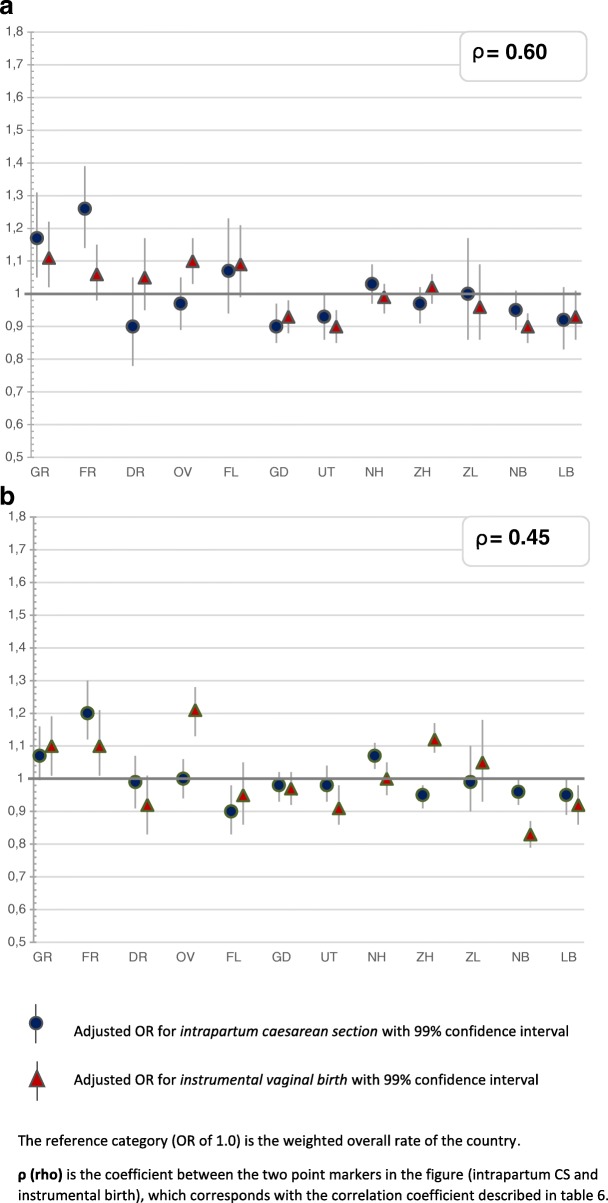
**a**: Regional variation of intrapartum CS and instrumental birth for women in midwife-led care. **b**: Regional variation of intrapartum CS and instrumental birth for women in obstetrician-led care

The greatest variation was found for the type of pain medication and whether a paediatrician was involved within 24 h after birth, followed by variation in augmentation after a spontaneous onset of labour. Less variation was found for induction of labour and prelabour CSs, and least for instrumental vaginal births and intrapartum CSs (Figs. 2, 3, 4, 5, 6 and 7). Similar variation in intervention rates was found for births in midwife-led care compared to those in obstetrician-led care at the onset of labour in the same region (Additional file 1: Table S5). The adverse neonatal and maternal outcomes were not lower in regions with higher intervention rates (Additional file 1: Table S8).

### Regional variations

Table 2 describes the intervention rates by region in subgroups stratified by parity, and Additional file 2: Table S4 the crude and adjusted ORs with confidence intervals, on which Figs. 2, 3, 4, 5, 6 and 7 are based. Most variation was found for the type of pain medication during labour (Fig. 2a and b), with epidural analgesia rates varying from between 12.3 to 37.5% in nulliparous and from between 4.6 to 13.8% in multiparous women, and rates of other pharmacological pain relief varying from between 14.8 to 43.0% in nulliparous and from between 9.8 to 26.8% in multiparous women without prelabour CS (Table 2). The variation of pain medication was similar for women in midwife-led compared to those in obstetrician-led care within the same region, with ρ = 0.97 (Additional file 1: Table S5), but rates were lower for women in midwife-led care. Generally, lower rates of other pharmacological pain relief were found in regions with higher rates of epidural analgesia, and vice versa. The correlation coefficient was ρ = − 0.61 for women in midwife-led care and ρ = − 0.68 in obstetrician-led care (Additional file 1: Table S6). There were no significant correlations between the use of pain medication and augmentation of labour, intrapartum oxytocin use, instrumental vaginal birth, intrapartum CS, or spontaneous vaginal birth (Additional file 1: Table S7). As can be seen from Fig. 3, considerable variation was found for the involvement of a paediatrician in the first 24 h after birth, with rates varying from between 36.9 to 60.3% for nulliparous and from between 25.6 to 42.7% for multiparous women (Table 2).

**Table 2:** Childbirth intervention rates by region total, and in subgroups by setting, stratified by parity (percentages)

Nulliparous women, total and by care setting at the onset of labour (abbreviated as ‘midwife’ or ‘obstetrician’)													
	Total	GR	FR	DR	OV	FL	GD	UT	NH	ZH	ZL	NB	LB
Total *n*													
* All women*	276,701	8901	9564	6785	18,051	7233	30,961	23,662	49,582	63,785	4853	38,544	14,780
* Midwife*	153,091	4827	5701	3550	10,292	3979	18,559	14,293	29,280	32,817	2695	20,2019	6879
* Obstetrician*	121,612	4045	3831	3217	7648	3230	12,247	9266	19,663	30,283	2140	18,198	7844
Induction of labour, %													
* All women*	21.2	24.8	20.7	26.2	23.3	20.8	18.6	18.0	19.1	23.0	22.4	21.4	24.4
* Obstetrician*	48.2	54.6	52.2	55.1	54.3	46.9	47.0	46.1	47.6	48.0	51.2	45.5	46.3
Augmentation after spontaneous onset of labour, *%*													
* All women*	42.9	40.7	38.0	41.5	36.2	48.4	44.3	45.9	43.7	42.1	33.5	45.6	42.2
* Midwife*	39.1	37.9	34.8	37.5	32.7	43.3	41.2	43.4	40.3	37.1	29.5	41.8	37.6
* Obstetrician*	54.5	49.9	51.5	53.9	48.9	62.6	55.1	54.7	55.6	54.9	47.1	56.0	52.0
Intrapartum oxytocin use, *%*													
* All women*	62.2	62.4	61.7	63.3	59.0	66.5	64.3	65.6	63.4	62.4	55.1	59.9	58.0
* Obstetrician*	61.6	64.6	64.4	67.5	60.9	69.5	63.2	63.7	62.1	60.9	56.5	58.7	58.4
Epidural, *%*													
* All women without prelabour CS*	27.4	25.4	20.1	12.3	27.7	13.7	27.6	31.0	22.3	27.3	19.1	37.5	36.4
* Midwife*	19.8	19.5	14.2	7.9	19.8	8.7	20.9	24.4	16.0	17.9	13.0	28.8	26.2
* Obstetrician without prelabour CS*	38.1	33.1	30.0	17.7	39.3	20.1	38.7	42.4	32.6	38.5	27.7	48.4	46.3
Other pharmacological pain relief, *%*													
* All women without prelabour CS*	21.6	20.1	20.6	38.2	16.2	43.0	17.6	14.8	24.6	22.3	25.6	17.2	27.9
* Midwife*	17.7	14.7	16.5	30.0	13.0	35.4	14.8	13.1	21.2	17.6	19.0	14.8	20.9
* Obstetrician without prelabour CS*	27.1	27.1	27.6	48.0	20.8	53.1	22.0	17.7	29.8	28.0	34.8	20.1	34.7
Spontaneous vaginal birth, *%*													
* All women*	65.2	62.4	64.0	66.0	64.1	64.6	67.4	66.8	64.6	64.1	66.2	66.2	64.9
* Midwife*	74.3	72.0	72.7	74.5	73.1	71.8	75.6	75.2	73.2	74.3	75.9	75.7	75.8
* Obstetrician*	53.6	51.2	51.2	56.6	51.9	55.9	54.9	53.9	51.8	53.0	53.9	55.4	55.4
Instrumental vaginal birth, *%*													
* All women (without prelabour CS)*	17.9	19.3	17.8	17.5	19.4	18.5	17.1	17.2	18.3	19.1	17.0	16.2	16.6
* Midwife*	17.0	18.5	17.1	17.7	18.3	18.5	16.4	16.3	17.5	17.3	15.6	15.9	15.9
* Obstetrician (without prelabour CS)*	19.2	20.2	18.9	17.4	21.1	18.5	18.2	18.7	19.7	21.1	18.9	16.5	17.3
Caesarean Section, *%*													
* All women*	17.8	19.3	19.0	17.2	17.4	17.6	16.3	16.7	17.9	17.7	17.6	18.5	19.4
Prelabour CS, *%*													
* All women*	4.5	4.6	4.7	4.1	4.4	3.6	4.1	4.2	4.3	4.4	4.6	5.2	5.8
* Obstetrician*	10.2	10.0	11.5	8.6	10.2	7.9	10.2	10.6	10.7	9.2	10.4	11.1	10.8
Intrapartum CS, *%*													
* All women (without prelabour CS)*	13.9	15.4	15.1	13.7	13.6	14.6	12.7	13.1	14.2	13.9	13.7	14.0	14.4
* Midwife*	8.6	9.6	10.2	7.7	8.5	9.7	7.9	8.5	9.3	8.4	8.5	8.3	8.2
* Obstetrician (without prelabour CS)*	21.2	23.0	23.3	20.9	21.2	21.0	20.7	21.1	22.3	20.5	21.0	21.2	20.6
Involvement paediatrician < 24 h, *%*													
* All women*	50.4	59.6	49.9	51.1	47.7	48.3	55.9	60.0	36.9	50.8	46.9	53.0	60.3
* Midwife*	38.1	45.6	37.9	37.2	34.8	38.1	43.4	49.0	26.9	36.5	32.5	42.5	47.0
* Obstetrician*	65.8	76.3	67.6	66.5	64.8	60.8	74.7	76.9	51.6	66.4	65.6	64.5	71.9
*Multiparous women, total and by care setting at the onset of labour (abbreviated as ‘midwife’ or ‘obstetrician’)*													
	Total	GR	FR	DR	OV	FL	GD	UT	NH	ZH	ZL	NB	LB
Total *n*													
* All women*	338,029	10,540	13,004	9090	24,818	10,228	40,325	29,231	56,366	75,788	6474	45,643	16,522
* Midwife*	174,918	5186	7200	4334	13,630	4963	22,842	16,308	30,470	37,206	3373	21,821	7585
* Obstetrician*	161,286	5331	5765	4735	11,070	5246	17,324	12,812	25,414	37,940	3082	23,705	8862
Induction of labour, %													
* All women*	19.7	23.8	19.0	25.4	20.2	22.2	17.7	16.6	18.0	21.1	20.6	20.1	21.2
* Obstetrician*	41.1	47.0	43.0	48.8	44.8	43.3	41.1	37.6	39.4	41.7	43.3	38.8	39.7
Augmentation after spontaneous onset of labour, *%*													
* All women*	17.3	14.5	14.8	18.0	14.0	22.6	17.2	16.1	17.9	18.4	12.4	18.8	16.6
* Midwife*	11.2	8.8	9.5	12.0	8.6	14.2	11.3	11.5	12.1	11.6	7.1	11.7	10.1
* Obstetrician*	33.2	29.3	32.5	33.2	30.9	41.3	34.3	29.4	34.6	33.9	27.2	33.8	30.2
Intrapartum oxytocin use, *%*													
* All women*	44.7	47.0	47.6	51.7	44.5	51.7	46.3	43.4	45.4	45.6	41.4	40.1	39.7
* Obstetrician*	45.8	50.3	50.0	54.2	46.6	54.3	47.8	43.9	45.8	45.9	42.9	41.0	41.5
Epidural, *%*													
* All women without prelabour CS*	9.6	8.9	7.0	4.6	8.4	4.9	9.3	10.4	8.0	9.7	7.6	13.8	13.8
* Midwife*	3.0	2.7	2.0	0.8	2.2	1.6	3.0	4.2	2.6	2.6	2.3	4.8	4.3
* Obstetrician without prelabour CS*	18.1	16.0	14.5	8.6	17.5	8.6	18.7	19.9	15.8	18.1	14.7	23.8	23.6
Other pharmacological pain relief, *%*													
* All women without prelabour CS*	14.5	12.4	14.3	22.9	11.7	26.8	11.2	9.8	14.5	15.1	14.4	15.0	19.9
* Midwife*	6.6	5.0	5.6	9.5	5.2	12.6	5.3	5.1	7.2	6.5	4.7	7.9	8.4
* Obstetrician without prelabour CS*	24.6	20.8	27.3	37.3	21.3	42.5	20.0	17.0	25.0	25.1	26.8	23.0	31.7
Spontaneous vaginal birth, *%*													
* All women*	83.7	82.9	82.5	83.8	83.6	84.9	86.1	84.2	82.8	83.4	83.9	83.6	81.7
* Midwife*	96.9	96.6	96.2	97.3	96.6	96.9	97.2	97.1	96.8	96.8	97.1	96.9	97.1
* Obstetrician*	69.3	69.7	65.2	71.5	67.5	73.6	71.5	67.9	66.0	70.1	69.4	71.1	68.6
Instrumental vaginal birth, *%*													
* All women (without prelabour CS)*	3.5	3.7	4.1	3.5	4.2	3.1	3.3	3.1	3.1	3.8	3.7	3.1	3.5
* Midwife*	1.8	1.7	2.1	1.7	2.2	1.6	1.6	1.7	1.6	1.9	1.8	1.7	1.7
* Obstetrician (without prelabour CS)*	5.7	6.0	7.0	5.5	7.2	4.8	5.9	5.3	5.4	6.0	6.1	4.6	5.4
Caesarean Section, *%*													
* All women*	13.2	13.7	13.8	13.0	12.6	12.2	10.8	12.9	14.3	13.2	12.7	13.6	15.2
Prelabour CS, *%*													
* All women*	7.8	7.9	7.8	7.7	7.7	7.2	5.8	8.0	8.5	7.9	7.7	8.2	9.8
* Obstetrician*	16.4	15.6	17.6	14.7	17.1	14.0	13.4	18.2	18.8	15.6	16.3	15.9	18.0
Intrapartum CS, *%*													
* All women (without prelabour CS)*	5.8	6.2	6.4	5.7	5.3	5.4	5.3	5.4	6.3	5.8	5.4	5.9	6.0
* Midwife*	1.3	1.7	1.6	1.0	1.2	1.5	1.2	1.2	1.5	1.4	1.1	1.4	1.1
* Obstetrician (without prelabour CS)*	11.6	11.4	13.8	10.7	11.4	9.6	11.5	11.8	13.4	11.0	11.2	10.9	11.0
Involvement paediatrician < 24 h, *%*													
* All women*	35.6	41.1	34.5	36.3	33.9	34.0	37.8	41.9	25.6	37.0	36.0	37.0	42.7
*Midwife*	14.6	16.6	14.2	13.7	13.1	14.8	16.4	19.6	9.2	14.4	11.7	16.8	19.2
*Obstetrician*	58.2	65.0	59.9	57.0	59.3	52.0	66.1	70.2	45.2	59.1	62.7	55.5	62.7

Percentage of missing data: 0.6 for midwife- or obstetrician-led care, 0.7% for spontaneous vaginal birth, 0.7% for instrumental birth, 0.7% for caesarean section, 0.8% for induction of labour, 0.6% for augmentation of labour, 0.0% for oxytocin use, 0.4% for epidural and other pharmacological pain relief, 0.3% for involvement paediatrician < 24 h

Figure 4 shows maps with variations of spontaneous birth rates, CS rates, and rates of intrapartum oxytocin between regions. Rates of intrapartum oxytocin, used for induction or augmentation of labour, were found of between 55.1 and 66.5% for nulliparous and of between 39.7 and 51.7% for multiparous women (Table 2), and varied significantly across regions (Fig. 4c). Rates of augmentation after a spontaneous onset of labour varied across regions from between 33.5 to 48.4% for nulliparous and from between 12.4 to 22.6% for multiparous women (Table 2). Instrumental vaginal birth rates were lower (ρ = − 0.61) and spontaneous vaginal birth rates were higher (ρ = 0.66; Additional file 1: Table S7) in regions where rates of augmentation of labour were higher. Variations in augmentation of labour are shown in Fig. 5.

Less variation was found for induction of labour, instrumental vaginal birth, and prelabour and intrapartum CS. Rates of prelabour CS were found of between 3.6 and 5.8% for all nulliparous and of between 5.8 and 9.8% for all multiparous women, and induction of labour rates of between 18.0 and 26.2% for all nulliparous and of between 16.6 and 25.4% for all multiparous women (Table 2). Figure 6 illustrates the ORs of prelabour CS and induction of labour. Regions with higher rates of prelabour CS had higher rates of intrapartum CS as well (ρ = 0.67), and lower rates of spontaneous vaginal births (ρ = − 0.62; Additional file 1: Table S7).

Compared to the other interventions, least variation was found for intrapartum CS and instrumental vaginal birth for women without prelabour CS (Fig. 7a and b). Intrapartum CS rates varied from between 12.7 to 15.4% (nulliparous women) and from between 5.3 to 6.4% (multiparous women), and instrumental birth rates varied from between 16.2 to 19.4% (nulliparous women) and from between 3.1 to 4.2% (multiparous women) (Table 2). For midwife-led care, regions with higher intrapartum CS rates had higher instrumental birth rates as well (ρ = 0.60), but this correlation was not significant in obstetrician-led care at the onset labour (ρ = 0.45; Additional file 1: Table S6). For all nulliparous women, a variation of spontaneous vaginal birth rates was found of between 62.4 and 67.4%, and for multiparous women, of between 81.7 and 86.1% (Table 2).

### Neonatal and maternal outcomes

The results of the multivariable analyses for the childbirth outcomes are described in Table 3. The overall incidence of antepartum and intrapartum stillbirth was 0.12% and of neonatal mortality up to 7 days 0.08%, but the adjusted ORs did not vary significantly between regions (Table 3; not shown in figures). Correlation coefficients were therefore not calculated for these outcomes. The incidence of Apgar score below 7 at 5 min varied significantly across regions from between 0.7 to 1.5%. For third and fourth degree perineal tear, incidences varied from between 1.8 to 3.2% and for PPH from between 3.7 to 6.9%. The only intervention and adverse outcome that were significantly correlated, were augmentation of labour after a spontaneous onset of labour and PPH (ρ = 0.87; Additional file 1: Table S8).

**Table 3:** Neonatal and maternal outcomes by region (percentages, crude and adjusted^a^ ORs, compared to weighted mean, with 99% CIs

	Total	GR	FR	DR	OV	FL	GD	UT	NH	ZH	ZL	NB	LB
	614,661	19,441	22,568	15,875	42,860	17,461	71,284	52,886	105,944	139,567	11,327	84,147	31,301
Antepartum and intrapartum stillbirth, *n (%)*	717 (0.12)	23 (0.12)	32 2(0.14)	17 (0.11)	42 (0.10)	27 (0.15)	97 (0.14)	62 (0.12)	126 (0.12)	159 (0.11)	11 (0.10)	88 (0.10)	33 (0.11)
Crude OR [99% CI]		1.02 [0.61–1.69]	1.22 [0.79–1.88]	0.92 [0.51–1.65]	0.84 [0.57–1.24]	1.33 [0.83–2.13]	1.17 [0.89–1.53]	1.01 [0.73–1.39]	1.02 [0.80–1.31]	0.98 [0.78–1.23]	0.83 [0.41–1.71]	0.90 [0.68–1.19]	0.90 [0.59–1.39]
aOR^a^ [99% CI]		1.04 [0.62–1.73]	1.26 [0.81–1.97]	0.93 [0.51–1.71]	0.90 [0.61–1.33]	1.41 [0.86–2.32]	1.13 [0.85–1.50]	1.07 [0.76–1.50]	0.97 [0.74–1.27]	0.97 [0.75–1.25]	0.75 [0.35–1.61]	0.85 [0.63–1.14]	0.89 [0.58–1.37]
Neonatal mortality up to 7 days, *n (%)*	471 (0.08)	14 (0.07)	13 (0.06)	12 (0.08)	29 (0.07)	14 (0.08)	72 (0.10)	42 (0.08)	85 (0.08)	98 (0.07)	12 (0.11)	52 (0.06)	28 (0.09)
Crude OR [99% CI]		0.93 [0.49–1.78]	0.75 [0.38–1.46]	0.98 [0.49–1.97]	0.88 [0.55–1.39]	1.04 [0.54–1.99]	1.31 [0.95–1.80]	1.03 [0.69–1.53]	1.04 [0.77–1.40]	0.91 [0.68–1.21]	1.37 [0.68–2.76]	0.80 [0.56–1.15]	1.16 [0.72–1.86]
aOR^a^ [99% CI]		0.93 [0.48–1.79]	0.76 [0.39–1.51]	1.06 [0.52–2.14]	0.83 [0.51–1.36]	1.04 [0.52–2.11]	1.34 [0.97–1.86]	1.02 [0.67–1.57]	1.01 [0.73–1.40]	0.92 [0.67–1.27]	1.38 [0.68–2.79]	0.80 [0.56–1.16]	1.08 [0.67–1.76]
Apgar score below 7 at 5 minutes^b^, *n (%)*	6410 (1.00)	291 (1.5)	280 (1.2)	132 (0.8)	354 (0.8)	163 (0.9)	715 (1.0)	460 (0.9)	1136 (1.1)	1735 (1.2)	83 (0.7)	777 (0.9)	284 (0.9)
Crude OR [99% CI]		1.53 [1.32–1.77]	1.26 [1.09–1.46]	0.84 [0.68–1.04]	0.84 [0.73–0.96]	0.95 [0.78–1.14]	1.02 [0.92–1.12]	0.88 [0.78–0.99]	1.09 [1.00–1.18]	1.26 [1.17–1.36]	0.74 [0.57–0.96]	0.94 [0.85–1.03]	0.92 [0.79–1.07]
aOR^a^ [99% CI]		1.47 [1.26–1.71]	1.28 [1.10–1.49]	0.90 [0.73–1.12]	0.85 [0.74–0.98]	0.96 [0.78–1.18]	1.05 [0.95–1.17]	0.88 [0.78–0.997]	0.99 [0.90–1.08]	1.18 [1.09–1.28]	0.78 [0.60–1.02]	0.94 [0.85–1.03]	0.92 [0.79–1.07]
3rd and 4th degree perineal tear for vaginal births, *%*	14,065 (2.76)	432 (2.68)	528 (2.84)	423 (3.15)	1061 (2.95)	369 (2.49)	1638 (2.68)	1369 (3.07)	2741 (3.13)	2725 (2.38)	173 (1.83)	1919 (2.79)	687 (2.73)
Crude OR [99% CI]		0.99 [0.88–1.12]	1.05 [0.94–1.17]	1.17 [1.04–1.32]	1.10 [1.01–1.19]	0.92 [0.81–1.04]	0.99 [0.93–1.06]	1.14 [1.06–1.23]	1.16 [1.10–1.23]	0.88 [0.83–0.93]	0.67 [0.56–0.81]	1.03 [0.97–1.10]	1.01 [0.92–1.11]
aOR^a^ [99% CI]		1.00 [0.88–1.12]	1.09 [0.98–1.22]	1.17 [1.03–1.33]	1.10 [1.01–1.91]	0.96 [0.83–1.10]	0.99 [0.92–1.06]	1.09 [1.01–1.18]	1.15 [1.08–1.22]	0.88 [0.82–0.93]	0.70 [0.58–0.84]	1.01 [0.95–1.08]	1.01 [0.92–1.11]
Postpartum haemorrhages ≥1000 ml, *%*	35,868 (6.00)	1088 (5.62)	1147 (5.17)	977 (6.19)	2283 (5.41)	1041 (5.99)	4610 (6.53)	3054 (5.85)	6155 (6.03)	7832 (5.79)	411 (3.72)	5430 (6.88)	1840 (5.95)
Crude OR [99% CI]		0.99 [0.91–1.06]	0.90 [0.84–0.97]	1.09 [1.01–1.18]	0.95 [0.90–0.998]	1.05 [0.97–1.14]	1.16 [1.11–1.20]	1.03 [0.98–1.08]	1.06 [1.02–1.10]	1.02 [0.98–1.05]	0.64 [0.57–0.72]	1.22 [1.17–1.27]	1.05 [0.99–1.11]
aOR^a^ [99% CI]		0.99 [0.92–1.07]	0.92 [0.85–0.99]	1.11 [1.02–1.21]	0.94 [0.89–0.995]	1.09 [0.998–1.18]	1.14 [1.09–1.19]	0.98 [0.93–1.03]	1.04 [0.99–1.08]	1.02 [0.99–1.06]	0.66 [0.58–0.74]	1.20 [1.15–1.25]	1.04 [0.98–1.10]

^a^Odds ratios, adjusted for parity, maternal age, ethnic background, socioeconomic status and urbanisation

^b^Antepartum and intrapartum stillbirth cases are excluded for analyses of Apgar score below 7 at 5 min

Percentage of missing data: 0.0% for antepartum and intrapartum stillbirth, 0.1% for neonatal mortality, 0.1% for Apgar score below 7 at 5 minutes, 1.4% for 3rd and 4th degree perineal tear, 2.7% for postpartum haemorrhages > 1000 ml

## Discussion

In this nationwide study, most interregional variation was found for the different types of pain medication (epidural analgesia or other pharmacological pain relief), and for the involvement of a paediatrician in the first 24 h after birth. Less variation was found for prelabour CS, augmentation and induction of labour, and least for instrumental vaginal birth and intrapartum CS rates. Regions with higher rates of one intervention did not have higher rates of all other interventions. Interventions that were correlated, were epidural analgesia and other pharmacological pain relief (negatively), augmentation of labour and instrumental vaginal birth (negatively), intrapartum CS and prelabour CS (positively), and for women in midwife-led care at the onset of labour, intrapartum CS and instrumental vaginal birth (positively). Regional variation was similar for women in midwife-led compared to those in obstetrician-led care within the same region. PPH occurred more often in regions where rates of augmentation of labour were higher. Antepartum and neonatal mortality rates did not vary significantly. Regions with higher intervention rates did not have lower rates of adverse neonatal and maternal outcomes, or vice versa.

### Limitations and strengths

This study is based on routinely collected data. Reporting bias is an issue in any register dataset, particularly for subjective outcomes, such as Apgar score and blood loss. Pitfalls in the use of these register-based data are described in a recent article of De Jonge et al. [44]. Misclassification is expected to be similar across regions and it is unlikely that it accounts for any of the variations. Another limitation is the absence or incompleteness of some variables in the dataset, such as maternal body mass index, congenital disorders, and obstetric history of low birth weight or previous CS. However, it is unlikely that this explains all variations observed, because adjustments for maternal characteristics did not lead to considerable changes in regional variation. Besides, it does not explain the large variation in pain medication and involvement of a paediatrician. On the other hand, regional variations in subgroups of different ethnic backgrounds could explain some of the variations. Secondly, regions with higher rates of referrals from midwife-led to obstetrician-led care, may have more low- or medium-risk women in obstetrician-led care, which might be reflected by lower intervention rates in obstetrician-led care, and higher rates in midwife-led care. However, our results showed strong positive correlations between intervention rates in midwife-led and obstetrician-led care within the same region. Last, by calculating correlation coefficients between regional adjusted ORs, it was not possible to account for the confidence intervals of the ORs. Therefore, these calculated correlations are only a rough indicator of relevant and significant correlations between variables. Besides, in case of minor variation in ORs, a Spearman’s rank correlation coefficient readily becomes insignificant, since it is based on ranking of the twelve regions. The Spearman’s rank correlation coefficients should be interpreted with caution, also because of multiple testing.

To our knowledge, this is the first study investigating regional variations of multiple interventions in childbirth in the Netherlands. A major strength of this study is its inclusion of almost all births in the Netherlands between 2010 and 2013. As stated in a Lancet series on Midwifery, available data strongly suggest an urgent need for more research to assess the appropriate use of interventions in childbirth [10]. This study contributes to this need. Because the results were described separately for women in midwife- and obstetrician-led care at the onset labour, it has become clearer in which subgroups variations in interventions are more prevalent. Another strength of this explorative study is the comparison of groups of births based on the mothers’ residential postal codes rather than her place of birth. Presence of a tertiary academic hospital in a region has had limited impact on results in this way, since in all regions both low- and high risk women are represented, women have access to all types of birth settings, while not all types are present in all regions, and confounders are more equally distributed than between hospitals [44]. However, other confounders, such as distance to a hospital, may still have influenced the outcomes.

Multilevel analyses were not performed, since the aim of this study was to explore regional variations that are not explained by maternal characteristics but may be explained by variations between care professionals and/or care settings (midwifery practices, hospitals).

### Interpretation and further research

The results from previous studies on regional variations in perinatal mortality and PPH in the Netherlands were not completely consistent with our results, probably due to older data and different samples [49, 51]. It is not possible to establish causal relationship in our study, for instance between augmentation of labour and severe PPH. However, the results are consistent with findings from previous studies that showed an association between oxytocin use during labour and severe PPH [52, 53]. Other studies showed greater variations between regions within a country than our study [29, 31–33, 54, 55]. Although variation in for instance augmentation of labour appears limited, an additional 10,300 nulliparous women would receive oxytocin for augmentation each year if the highest regional rate would become the national rate, compared to the lowest rate. Even in case of limited variation in intervention rates, crude numbers show that variation might nonetheless be unwarranted. An aim of evidence-based practice is to minimize unwarranted variation in the use of interventions [56, 57]. However, it is still unknown what would be the best rate for augmentation of labour and for other interventions. Regions with higher rates of augmentation of labour had on one hand higher rates of PPH, but on the other hand lower instrumental vaginal birth rates. Whether there is a causal relationship between these variables, needs to be investigated in further research. Generally, the optimal rate is the lowest rate with comparable neonatal and maternal outcomes. In our study adverse neonatal and maternal outcomes were not lower in regions with higher intervention rates. However, achieving a low intervention rate should not be an aim in itself [10, 57]. It is not possible to identify the optimal rate of interventions based on this study. An essential element in improving quality of care, is that care providers critically audit remarkably high and low rates [10, 58]. This study intends to contribute to this debate. Following national guidelines and using the recommendations of the WHO might help in achieving the optimal use of interventions [15–17, 23, 58].

On the other hand, differences in regional guidelines and in adherence to national guidelines may explain a part of the large variation in type of pain medication and involvement of a paediatrician. Use of epidural analgesia for women with a single fetus in cephalic position after 37 weeks’ gestation, has almost tripled between 2000 and 2009 in the Netherlands (from 7.7 to 21.9%) [59]. In 2008, a multidisciplinary guideline on pain medication was published, in which adequate pain relief upon request for all women during labour was advised, with epidural analgesia as the most effective method for pain relief. Two randomized controlled trials showed that women were more satisfied with epidural analgesia compared to patient-controlled remifentanil [60, 61], but access to pain medication should not be at the expense of continuous support, which can reduce the need for pain medication [22]. The large variation in rates of pain medication suggests different degrees of implementation of evidence and national guidelines, leading to disparity in accessibility to pain medication. Furthermore, the absence of a national guideline on when a paediatrician needs to be involved after birth and differences in accessibility may explain a part of the large variation in the rates of paediatric involvement, leading to differences in care and costs. Further research is required to examine which medical and non-medical factors may explain the large variations in pain medication and involvement of a paediatrician.

Clinical practice is influenced by characteristics of the care provider, such as age, educational background, perceptions of risks, and views on childbirth [62–66]. Culture within the work environment may encourage care providers to take similar decisions, and variations are therefore not merely individual [67]. Differences in perceptions and attitudes may result in differences in local practice and guidelines. The fact that variations were found between regions, even after adjustments for maternal characteristics, suggests that there may be cultural differences between regions, reflected in differences in the views of care providers on childbirth [63, 68, 69]. The large variation, in particular for pain medication and involvement of a paediatrician, cannot be explained by clinical variations only. Similarities in variations in interventions that were found between women in midwife-led and obstetrician-led care, suggest similar practice by midwives and obstetricians within regions. These similarities existed in interventions with minor variation as well as in those with considerable variation. The results of this study call for implementation of evidence-based interventions, and for investigation into indications for the use of interventions in childbirth [10]. The Robson Classification System could be used to explore subgroups of women that account for the greatest variation [70]. Limited variation in some of the interventions in our study may indicate consensus about its use. However, variations may be greater between midwifery practices, hospitals, collaborations or care providers, than between regions where variations between organisations and practitioners will have been averaged. In further research, variations within the regions should therefore be investigated.

## Conclusions

The greatest variation was found for the type of pain medication and the involvement of a paediatrician, and the least for instrumental vaginal birth and intrapartum CS rates. The rates of adverse outcomes were not lower in regions with higher intervention rates. Care providers should critically audit remarkable variations, since these may be unwarranted. Variation may be explained to some extent by a difference in the degree of implementation of national guidelines between regions. Further research should therefore focus on variations in evidence-based interventions and indications for the use of interventions in childbirth.

## Additional files

Additional file 1: Tables with correlations within and between interventions and obstetric outcomes tested with Spearman’s rho: **Table S5**. Correlations within interventions among women in midwife-led and interventions among women in obstetrician-led care at the onset of labour; **Table S6**. Correlations between interventions in subgroups of women in midwife- or obstetrician-led care at the onset of labour; **Table S7**. Correlations between interventions; **Table S8**. Correlations between interventions and obstetric outcomes (DOCX 22 kb)

Additional file 2: A table with multivariable logistic regression of intervention rates by region, in the following subgroups: all women; women in midwife-led care at the onset labour; women in obstetrician-led care at the onset of labour. **Table S4**: Crude and adjusted* ORs of childbirth interventions by region, compared to the weighted mean, with 99% CIs (DOCX 59 kb)

## Abbreviations

CS: Caesarean section
OR: Odds ratio
PPH: Postpartum haemorrhage of 1000 ml or more
SES: Socioeconomic status
VU: Vrije Universiteit
WHO: World Health Organization

## References

[1] EURO-PERISTAT Project with SCPE and EUROCAT. European Perinatal Health Report. Health and care of pregnant women and babies in Europe in 2010. EURO-PERISTAT; 2013. https://www.europeristat.com.

[2] Lumbiganon P, Laopaiboon M, Gulmezoglu AM, Souza JP, Taneepanichskul S, Ruyan P (2010). Method of delivery and pregnancy outcomes in Asia: the WHO global survey on maternal and perinatal health 2007-08. Lancet.

[3] Notzon FC (1990). International differences in the use of obstetric interventions. JAMA.

[4] Vogel JP, Gulmezoglu AM, Hofmeyr GJ, Temmerman M (2014). Global perspectives on elective induction of labor. Clin Obstet Gynecol.

[5] Dublin S, Johnson KE, Walker RL, Avalos LA, Andrade SE, Beaton SJ (2014). Trends in elective labor induction for six United States health plans, 2001-2007. J Women's Health (Larchmt).

[6] Martin JA, Hamilton BE, Osterman MJ (2014). Births in the United States, 2013. NCHS Data Brief.

[7] Osterman MJ, Martin JA (2014). Recent declines in induction of labor by gestational age. NCHS Data Brief.

[8] Cavallaro FL, Cresswell JA, Franca GV, Victora CG, Barros AJ, Ronsmans C (2013). Trends in caesarean delivery by country and wealth quintile: cross-sectional surveys in southern Asia and sub-Saharan Africa. Bull World Health Organ.

[9] Patterson JA, Roberts CL, Ford JB, Morris JM (2011). Trends and outcomes of induction of labour among nullipara at term. Aust N Z J Obstet Gynaecol.

[10] Miller S, Abalos E, Chamillard M, Ciapponi A, Colaci D, Comande D (2016). Beyond too little, too late and too much, too soon: a pathway towards evidence-based, respectful maternity care worldwide. Lancet.

[11] Kilsztajn S, Carmo MS, Machado LC, Lopes ES, Lima LZ (2007). Caesarean sections and maternal mortality in Sao Paulo. Eur J Obstet Gynecol Reprod Biol.

[12] Renfrew MJ, McFadden A, Bastos MH, Campbell J, Channon AA, Cheung NF (2014). Midwifery and quality care: findings from a new evidence-informed framework for maternal and newborn care. Lancet.

[13] Tracy SK, Sullivan E, Wang YA, Black D, Tracy M (2007). Birth outcomes associated with interventions in labour amongst low risk women: a population-based study. Women Birth.

[14] Villar J, Valladares E, Wojdyla D, Zavaleta N, Carroli G, Velazco A (2006). Caesarean delivery rates and pregnancy outcomes: the 2005 WHO global survey on maternal and perinatal health in Latin America. Lancet.

[15] World Health Organization (1996). Care in Normal Birth: a practical guide.

[16] World Health Organization (2011). WHO recommendations for induction of labour.

[17] World Health Organization (2014). WHO recommendations for augmentation of labour.

[18] Bernitz S, Oian P, Rolland R, Sandvik L, Blix E (2014). Oxytocin and dystocia as risk factors for adverse birth outcomes: a cohort of low-risk nulliparous women. Midwifery.

[19] Mozurkewich E, Chilimigras J, Koepke E, Keeton K, King VJ (2009). Indications for induction of labour: a best-evidence review. BJOG.

[20] Anim-Somuah M, Smyth RM, Jones L (2011). Epidural versus non-epidural or no analgesia in labour. Cochrane Database Syst Rev.

[21] Hawkins JL (2010). Epidural analgesia for labor and delivery. N Engl J Med.

[22] Hodnett ED, Gates S, Hofmeyr GJ, Sakala C, Weston J (2011). Continuous support for women during childbirth. Cochrane Database Syst Rev.

[23] Betran AP, Torloni MR, Zhang JJ, Gulmezoglu AM (2015). WHO statement on caesarean section rates. BJOG.

[24] Althabe F, Sosa C, Belizan JM, Gibbons L, Jacquerioz F, Bergel E (2006). Cesarean section rates and maternal and neonatal mortality in low-, medium-, and high-income countries: an ecological study. Birth.

[25] Nippita TA, Lee YY, Patterson JA, Ford JB, Morris JM, Nicholl MC (2015). Variation in hospital caesarean section rates and obstetric outcomes among nulliparae at term: a population-based cohort study. BJOG.

[26] Sandall J, Soltani H, Gates S, Shennan A, Devane D (2013). Midwife-led continuity models versus other models of care for childbearing women. Cochrane Database Syst Rev.

[27] Althabe F, Buekens P, Bergel E, Belizan JM, Campbell MK, Moss N (2008). A behavioral intervention to improve obstetrical care. N Engl J Med.

[28] Von Dadelszen P, Sawchuck D, McMaster R, Douglas MJ, Lee SK, Saunders S (2010). The active implementation of pregnancy hypertension guidelines in British Columbia. Obstet Gynecol.

[29] Bragg F, Cromwell DA, Edozien LC, Gurol-Urganci I, Mahmood TA, Templeton A (2010). Variation in rates of caesarean section among English NHS trusts after accounting for maternal and clinical risk: cross sectional study. BMJ.

[30] Chalmers B, Kaczorowski J, Levitt C, Dzakpasu S, O'Brien B, Lee L (2009). Use of routine interventions in vaginal labor and birth: findings from the maternity experiences survey. Birth.

[31] Chalmers B, Kaczorowski J, O’Brien B, Royle C (2012). Rates of interventions in labor and birth across Canada: findings of the Canadian maternity experiences survey. Birth.

[32] Lutomski JE, Morrison JJ, Lydon-Rochelle MT (2012). Regional variation in obstetrical intervention for hospital birth in the Republic of Ireland, 2005-2009. BMC Pregnancy Childbirth.

[33] Mikolajczyk RT, Schmedt N, Zhang J, Lindemann C, Langner I, Garbe E (2013). Regional variation in caesarean deliveries in Germany and its causes. BMC Pregnancy Childbirth.

[34] Elferink-Stinkens PM, Brand R, Le Cessie S, Van Hemel OJ (1996). Large differences in obstetrical intervention rates among Dutch hospitals, even after adjustment for population differences. Eur J Obstet Gynecol Reprod Biol.

[35] Heres MH, Pel M, Elferink-Stinkens PM, Van Hemel OJ, Treffers PE (1995). The Dutch obstetric intervention study--variations in practice patterns. Int J Gynaecol Obstet.

[36] Brownlee S, Chalkidou K, Doust J, Elshaug AG, Glasziou P, Heath I (2017). Evidence for overuse of medical services around the world. Lancet.

[37] Stichting Perinatale Registratie Nederland (2014). PRN Koppelingsprocedure 2013 (LVR1, LVRh, LVR2 en LNR-deelregistraties).

[38] Tromp M, Ravelli AC, Meray N, Reitsma JB, Bonsel GJ (2008). An efficient validation method of probabilistic record linkage including readmissions and twins. Methods Inf Med.

[39] Stichting Perinatale Registratie Nederland (2013). Perinatale Registratie Nederland Grote Lijnen 1999–2012.

[40] Hitzert M, Hermus MM, Boesveld II, Franx A, Van der Pal-de Bruin KK, Steegers EE (2017). Cost-effectiveness of planned birth in a birth Centre compared with alternative planned places of birth: results of the Dutch birth Centre study. BMJ Open.

[41] Hitzert M, Hermus MA, Scheerhagen M, Boesveld IC, Wiegers TA, Van den Akker-van Marle ME (2016). Experiences of women who planned birth in a birth Centre compared to alternative planned places of birth. Results of the Dutch birth Centre study. Midwifery.

[42] Amelink-Verburg MP, Rijnders ME, Buitendijk SE (2009). A trend analysis in referrals during pregnancy and labour in Dutch midwifery care 1988-2004. BJOG.

[43] Offerhaus PM, De Jonge A, Van der Pal-De Bruin KM, Hukkelhoven CW, Scheepers PL, Lagro-Janssen AL (2014). Change in primary midwife-led care in the Netherlands in 2000-2008: a descriptive study of caesarean sections and other interventions among 789,795 low risk births. Midwifery.

[44] De Jonge A, Wouters M, Klinkert J, Brandenbarg J, Zwart JJ, Van DJ (2017). Pitfalls in the use of register-based data for comparing adverse maternal and perinatal outcomes in different birth settings. BJOG.

[45] Schuurhuis A, Roumen FJ, De Boer JB (2009). Practice guideline ‘Pharmaceutical pain treatment during labour’; the woman’s request is sufficient indication. Ned Tijdschr Geneeskd.

[46] Bayrampour H, Salmon C, Vinturache A, Tough S (2015). Effect of depressive and anxiety symptoms during pregnancy on risk of obstetric interventions. J Obstet Gynaecol Res.

[47] De Jonge A, Geerts CC, Van der Goes BY, Mol BW, Buitendijk SE, Nijhuis JG (2015). Perinatal mortality and morbidity up to 28 days after birth among 743 070 low-risk planned home and hospital births: a cohort study based on three merged national perinatal databases. BJOG.

[48] Offerhaus PM, Geerts C, De Jonge A, Hukkelhoven CW, Twisk JW, Lagro-Janssen AL (2015). Variation in referrals to secondary obstetrician-led care among primary midwifery care practices in the Netherlands: a nationwide cohort study. BMC Pregnancy Childbirth.

[49] Tromp M, Eskes M, Reitsma JB, Erwich JJ, Brouwers HA, Rijninks-van Driel GC (2009). Regional perinatal mortality differences in the Netherlands; care is the question. BMC Public Health.

[50] The BMJ. 11. Correlation and regression. http://www.bmj.com/about-bmj/resources-readers/publications/statistics-square-one/11-correlation-and-regression. Accessed 11 Jan 2018.

[51] Prick BW, Auf Altenstadt JF, Hukkelhoven CW, Bonsel GJ, Steegers EA, Mol BW (2015). Regional differences in severe postpartum hemorrhage: a nationwide comparative study of 1.6 million deliveries. BMC Pregnancy Childbirth.

[52] Belghiti J, Kayem G, Dupont C, Rudigoz RC, Bouvier-Colle MH, Deneux-Tharaux C (2011). Oxytocin during labour and risk of severe postpartum haemorrhage: a population-based, cohort-nested case-control study. BMJ Open.

[53] Sheiner E, Sarid L, Levy A, Seidman DS, Hallak M (2005). Obstetric risk factors and outcome of pregnancies complicated with early postpartum hemorrhage: a population-based study. J Matern Fetal Neonatal Med.

[54] Rabilloud M, Ecochard R, Guilhot J, Toselli A, Mabriez JC, Matillon Y (1998). Study of the variations of the cesarean sections rate in the Rhone-Alpes region (France): effect of women and maternity service characteristics. Eur J Obstet Gynecol Reprod Biol.

[55] Hanley GE, Janssen PA, Greyson D (2010). Regional variation in the cesarean delivery and assisted vaginal delivery rates. Obstet Gynecol.

[56] Wennberg JE, Fisher ES, Skinner JS. Geography and the debate over Medicare reform. Health Aff (Millwood) 2002, Suppl Web Exclusives: W96–114. 10.1377/hlthaff.w2.9. 10.1377/hlthaff.w2.96 12703563

[57] Goodman DC (2009). Unwarranted variation in pediatric medical care. Pediatr Clin N Am.

[58] Glantz JC (2012). Obstetric variation, intervention, and outcomes: doing more but accomplishing less. Birth.

[59] Wassen MM, Hukkelhoven CW, Scheepers HC, Smits LJ, Nijhuis JG, Roumen FJ (2014). Epidural analgesia and operative delivery: a ten-year population-based cohort study in the Netherlands. Eur J Obstet Gynecol Reprod Biol.

[60] Freeman LM, Bloemenkamp KW, Franssen MT, Papatsonis DN, Hajenius PJ, Hollmann MW (2015). Patient controlled analgesia with remifentanil versus epidural analgesia in labour: randomised multicentre equivalence trial. BMJ.

[61] Logtenberg S, Oude Rengerink K, Verhoeven CJ, Freeman LM, Van den Akker E, Godfried MB (2017). Labour pain with remifentanil patient-controlled analgesia versus epidural analgesia: a randomised equivalence trial. BJOG.

[62] Burns LR, Geller SE, Wholey DR (1995). The effect of physician factors on the cesarean section decision. Med Care.

[63] Healy S, Humphreys E, Kennedy C (2016). Midwives’ and obstetricians’ perceptions of risk and its impact on clinical practice and decision-making in labour: an integrative review. Women Birth.

[64] Croskerry P (2002). Achieving quality in clinical decision making: cognitive strategies and detection of bias. Acad Emerg Med.

[65] Eldenburg L, Waller WS (2001). Decision-case mix model for analyzing variation in cesarean rates. Med Decis Mak.

[66] Styles M, Cheyne H, O'Carroll R, Greig F, Dagge-Bell F, Niven C (2011). The development of research tools used in the STORK study (the Scottish trial of refer or keep) to explore midwives' intrapartum decision making. Midwifery.

[67] De Jong JD, Groenewegen PP, Westert GP (2003). Mutual influences of general practitioners in partnerships. Soc Sci Med.

[68] Pel M, Heres MH, Hart AA, Van der Veen F, Treffers PE (1995). Provider-associated factors in obstetric interventions. Eur J Obstet Gynecol Reprod Biol.

[69] Mead MM, Kornbrot D (2004). The influence of maternity units’ intrapartum intervention rates and midwives’ risk perception for women suitable for midwifery-led care. Midwifery.

[70] Zhang J, Geerts C, Hukkelhoven C, Offerhaus P, Zwart J, De Jonge A (2016). Caesarean section rates in subgroups of women and perinatal outcomes. BJOG.

