# Supplementary material for: Regional variations in childbirth interventions in the Netherlands: a nationwide explorative study
Source: BMC Pregnancy Childbirth. 2018 Jun 1;18:192. doi: 10.1186/s12884-018-1795-0 (PMC5984340; doi:10.1186/s12884-018-1795-0)
Supplement: Supplementary file 1 — Tables with correlations within and between interventions and obstetric outcomes tested with Spearman’s rho: Table S5. Correlations within interventions among women in midwife-led and interventions among women in obstetrician-led care at the onset of labour; Table S6. Correlations between interventions in subgroups of women in midwife- or obstetrician-led care at the onset of labour; Table S7. Correlations between interventions; Table S8. Correlations between interventions and obstetric outcomes (DOCX 22 kb) [file 12884_2018_1795_MOESM1_ESM.docx]

**Additional file 1: Correlations within and between interventions and obstetric outcomes tested with Spearman’s rho (two-tailed significance level)**

All correlations are based on adjusted OR’s of the intervention rates of the region (adjusted for parity, maternal age, ethnic background, socioeconomic status and urbanisation). The correlations are therefore on region level and not on individual women level.

A p-value of 0.05 corresponds with a correlation of ρ ≥ 0.57 or ≤ - 0.57 (95% confidence intervals 0.001-0.86).

Since the sample size for all measured correlations is the same, namely 12 regions, the correlation is significant at the same value of ρ for all measured correlations. Correlations with ρ ≥ 0.60 or ≤ - 0.60 are indicated in bold type, since they are considered strong.

|  | | **Interventions among women in midwife-led care at onset of labour** | | | | | |
| --- | --- | --- | --- | --- | --- | --- | --- |
|  |  | *Augmentation after spontaneous onset of labour* | *Epidural** | *Other pharmacological pain relief** | *Instrumental vaginal birth** | *Intrapartum CS** | *Involvement paediatrician <24 hours* |
| **Interventions among women in obstetrician-led care at onset of labour** | *Augmentation after spontaneous onset of labour* | **ρ = 0.76** |  |  |  |  |  |
|  | *Epidural** |  | **ρ = 0.97** |  |  |  |  |
|  | *Other pharmacological pain relief** |  |  | **ρ = 0.97** |  |  |  |
|  | *Instrumental vaginal birth** |  |  |  | **ρ = 0.70** |  |  |
|  | *Intrapartum CS** |  |  |  |  | ρ = 0.43 |  |
|  | *Involvement paediatrician <24 hours* |  |  |  |  |  | **ρ = 0.67** |

**Table S5. Correlations within interventions among women in midwife-led and interventions among women in obstetrician-led care at onset of labour**

*Measured in a group of women without prelabour CS.

**Table S6. Correlations between interventions in subgroups of women in midwife- or obstetrician-led care at onset of labour**

|  | | **Interventions among women in midwife-led care at onset of labour** | | **Interventions among women in**  **obstetrician-led care at onset of labour** | |
| --- | --- | --- | --- | --- | --- |
|  |  | *Epidural* | *Instrumental vaginal birth* | *Epidural** | *Instrumental vaginal birth** |
| **Interventions among women in midwife-led care at onset of labour** | *Other pharmacological pain relief* | **ρ = - 0.61** |  |  |  |
|  | *Intrapartum CS* |  | **ρ = 0.60** |  |  |
| **Interventions among women in**  **obstetrician-led care at onset of labour** | *Other pharmacological pain relief** |  |  | **ρ = - 0.68** |  |
|  | *Intrapartum CS** |  |  |  | ρ = 0.45 |

*Measured in a group of women without prelabour CS.

|  | ***Induction of labour*** | ***Augmentation after spontaneous onset of labour*** | ***Intrapartum oxytocin use*** | ***Epidural**** | ***Other pharmacological pain relief**** | ***Instrumental vaginal birth**** | ***Prelabour CS*** | ***Involvement paediatrician <24 hrs*** |
| --- | --- | --- | --- | --- | --- | --- | --- | --- |
| ***Induction of labour*** |  | ρ = 0.01 |  | ρ = - 0.39 | ρ = 0.55 | ρ = 0.56 |  | ρ = 0.07 |
| ***Augmentation after spontaneous onset of labour*** |  |  |  | ρ = 0.10 | ρ = 0.24 | **ρ = - 0.61** |  | ρ = 0.25 |
| ***Intrapartum oxytocin use*** |  |  |  | ρ = - 0.49 | ρ = 0.10 | ρ = 0.09 |  | ρ = 0.03 |
| ***Epidural**** |  |  |  |  | **ρ = - 0.61** | ρ = - 0.50 |  | ρ = 0.56 |
| ***Other pharmacological pain relief**** |  |  |  |  |  | ρ = 0.08 |  | ρ = - 0.22 |
| ***Instrumental vaginal birth**** |  |  |  |  |  |  |  | ρ = - 0.39 |
| ***Prelabour CS*** | ρ = 0.19 |  |  |  |  |  |  | ρ = 0.28 |
| ***Intrapartum CS**** | ρ = 0.48 | ρ = - 0.23 | ρ = - 0.02 | ρ = - 0.42 | ρ = 0.52 | ρ = 0.29 | **ρ = 0.67** | ρ = 0.12 |
| ***Spontaneous vaginal birth*** | ρ = - 0.54 | **ρ = 0.66** | ρ = 0.25 | ρ = 0.20 | ρ = - 0.18 | **ρ = - 0.72** | **ρ = - 0.62** | ρ = - 0.06 |

**Table S7. Correlations between interventions**

*Measured in a group of women without prelabour CS.

**Table S8. Correlations between interventions and obstetric outcomes**

|  | | **Obstetric outcomes** | | |
| --- | --- | --- | --- | --- |
|  |  | *Apgar score below 7 at 5 minutes* | *3^rd^ and 4^th^ degree perineal tear for vaginal births* | *Postpartum haemorrhages ≥1000 ml* |
| **Interventions among all women** | *Induction of labour* | ρ = 0.11 | ρ = - 0.12 | ρ = 0.09 |
|  | *Augmentation after spontaneous onset of labour* | ρ = 0.05 | ρ = 0.09 | **ρ = 0.87** |
|  | *Intrapartum oxytocin use* | ρ = 0.41 | ρ = 0.16 | ρ = 0.35 |
|  | *Epidural** | ρ = - 0.10 | ρ = 0.02 | ρ = 0.22 |
|  | *Other pharmacological pain relief** | ρ = - 0.04 | ρ = - 0.14 | ρ = 0.20 |
|  | *Spontaneous vaginal birth* | ρ = - 0.29 | ρ = 0.00 | ρ = 0.55 |
|  | *Instrumental vaginal birth** | ρ = 0.24 | ρ = - 0.11 | ρ = - 0.55 |
|  | *Caesarean section* | ρ = 0.20 | ρ = 0.09 | ρ = 0.16 |
|  | *Involvement paediatrician <24 hours* | ρ = 0.17 | ρ = - 0.13 | ρ = 0.24 |

*Measured in a group of women without prelabour CS.
