# Supplementary material for: Regional variations in childbirth interventions in the Netherlands: a nationwide explorative study
Source: BMC Pregnancy Childbirth. 2018 Jun 1;18:192. doi: 10.1186/s12884-018-1795-0 (PMC5984340; doi:10.1186/s12884-018-1795-0)
Supplement: Supplementary file 2 — A table with multivariable logistic regression of intervention rates by region, in the following subgroups: all women; women in midwife-led care at the onset labour; women in obstetrician-led care at the onset of labour. Table S4: Crude and adjusted* ORs of childbirth interventions by region, compared to the weighted mean, with 99% CIs (DOCX 59 kb) [file 12884_2018_1795_MOESM2_ESM.docx]

**Additional file 2. Table S4. Crude and adjusted* ORs of childbirth interventions by region, compared to the weighted mean, with 99% CIs**

|  | **GR** | **FR** | **DR** | **OV** | **FL** | **GD** | **UT** | **NH** | **ZH** | **ZL** | **NB** | **LB** |
| --- | --- | --- | --- | --- | --- | --- | --- | --- | --- | --- | --- | --- |
| **Total *n*** | 19,441 | 22,568 | 15,875 | 42,869 | 17,461 | 71,286 | 52,893 | 105,948 | 139,573 | 11,327 | 84,187 | 31,302 |
| **Induction of labour**  Crude OR [99% CI]  aOR* [99% CI] | 1.20 [1.16-1.25]  1.18 [1.13-1.23] | 0.92 [0.89-0.96]  0.90 [0.86-0.94] | 1.30 [1.25-1.36]  1.29 [1.23-1.35] | 1.03 [1.001-1.06]  1.01 [0.98-1.04] | 1.04 [0.99-1.08]  1.09 [1.04-1.15] | 0.83 [0.81-0.85]  0.82 [0.80-0.84] | 0.78 [0.76-0.80]  0.78 [0.76-0.80] | 0.85 [0.83-0.87]  0.88 [0.86-0.90] | 1.06 [1.04-1.08]  1.11 [1.08-1.13] | 1.02 [0.97-1.08]  1.00 [0.95-1.06] | 0.98 [0.96-1.004]  0.99 [0.97-1.02] | 1.10 [1.07-1.14]  1.08 [1.05-1.12] |
| **Augmentation of labour after spontaneous onset**  Crude OR [99% CI]  aOR* [99% CI] | 0.94 [0.90-0.99]  0.95 [0.90-0.996] | 0.85 [0.81-0.89]  0.92 [0.88-0.97] | 1.02 [0.97-1.07]  1.10 [1.04-1.17] | 0.79 [0.76-0.81]  0.80 [0.77-0.83] | 1.31 [1.25-1.37]  1.30 [1.24-1.37] | 1.06 [1.03-1.08]  1.09 [1.06-1.12] | 1.09 [1.06-1.12]  1.05 [1.02-1.09] | 1.12 [1.10-1.15]  1.00 [0.98-1.03] | 1.07 [1.05-1.09]  0.99 [0.97-1.01] | 0.71 [0.66-0.76]  0.74 [0.69-0.79] | 1.17 [1.14-1.20]  1.16 [1.13-1.19] | 1.04 [1.01-1.08]  1.02 [0.98-1.06] |
| **Intrapartum oxytocin use**  Crude OR [99% CI]  aOR* [99% CI] | 1.05 [1.01-1.10]  1.06 [1.01-1.10] | 1.03 [0.99-1.08]  1.05 [1.01-1.10] | 1.16 [1.11-1.21]  1.19 [1.13-1.25] | 0.92 [0.89-0.95]  0.92 [0.89-0.95] | 1.22 [1.17-1.28]  1.24 [1.19-1.30] | 1.07 [1.05-1.10]  1.07 [1.04-1.10] | 1.05 [1.02-1.08]  1.05 [1.02-1.08] | 1.05 [1.03-1.07]  1.04 [1.02-1.06] | 1.02 [1.002-1.04]  1.02 [0.998-1.04] | 0.80 [0.76-0.85]  0.79 [0.75-0.84] | 0.87 [0.85-0.89]  0.86 [0.85-0.88] | 0.84 [0.81-0.87]  0.82 [0.79-0.84] |
| **Epidural** *(women without prelabour CS)*  Crude OR [99% CI]  aOR* [99% CI] | 1.08 [1.03-1.14]  1.08 [1.03-1.14] | 0.79 [0.75-0.83]  0.84 [0.80-0.89] | 0.48 [0.44-0.51]  0.50 [0.46-0.54] | 1.09 [1.06-1.13]  1.15 [1.11-1.19] | 0.52 [0.49-0.56]  0.48 [0.45-0.52] | 1.16 [1.13-1.19]  1.19 [1.16-1.23] | 1.35 [1.31-1.39]  1.35 [1.30-1.39] | 0.95 [0.93-0.98]  0.86 [0.83-0.88] | 1.20 [1.17-1.22]  1.09 [1.06-1.12] | 0.80 [0.74-0.85]  0.84 [0.78-0.90] | 1.75 [1.71-1.80]  1.80 [1.76-1.85] | 1.73 [1.67-1.79]  1.77 [1.71-1.83] |
| **Other pharmacological pain relief** *(women without prelabour CS)*  Crude OR [99% CI]  aOR* [99% CI] | 0.82 [0.79-0.87]  0.82 [0.78-0.86] | 0.89 [0.86-0.93]  0.93 [0.89-0.97] | 1.81 [1.73-1.89]  1.87 [1.78-1.95] | 0.69 [0.66-0.71]  0.69 [0.67-0.72] | 2.19 [2.11-2.28]  2.14 [2.05-2.23] | 0.72 [0.70-0.74]  0.73 [0.71-0.75] | 0.60 [0.58-0.62]  0.58 [0.56-0.60] | 1.04 [1.02-1.06]  1.03 [1.001-1.05] | 1.00 [0.98-1.02]  0.97 [0.95-0.99] | 1.04 [0.98-1.10]  1.06 [1.001-1.13] | 0.82 [0.80-0.84]  0.82 [0.80-0.84] | 1.31 [1.27-1.36]  1.30 [1.26-1.35] |
| **Spontaneous vaginal birth**  Crude OR [99% CI]  aOR* [99% CI] | 0.90 [0.87-0.94]  0.89 [0.85-0.93] | 0.96 [0.92-0.995]  0.90 [0.87-0.94] | 1.04 [0.996-1.09]  1.00 [0.95-1.05] | 0.99 [0.97-1.02]  0.97 [0.94-0.999] | 1.06 [1.01-1.11]  1.03 [0.98-1.08] | 1.15 [1.12-1.18]  1.17 [1.14-1.20] | 1.06 [1.03-1.08]  1.12 [1.09-1.16] | 0.94 [0.92-0.96]  1.01 [0.99-1.03] | 0.96 [0.94-0.97]  0.97 [0.95-0.99] | 1.05 [0.99-1.11]  0.99 [0.94-1.05] | 1.01 [0.99-1.03]  1.05 [1.02-1.07] | 0.92 [0.89-0.94]  0.94 [0.91-0.98] |
| **Instrumental vaginal birth** *(women without prelabour CS)*  Crude OR [99% CI]  aOR* [99% CI] | 1.11 [1.05-1.18]  1.11 [1.05-1.18] | 1.00 [0.95-1.06]  1.07 [1.004-1.13] | 0.96 [0.90-1.03]  1.00 [0.93-1.07] | 1.09 [1.04-1.13]  1.14 [1.09-1.19] | 0.97 [0.91-1.03]  1.03 [0.96-1.11] | 0.93 [0.90-0.97]  0.93 [0.90-0.97] | 0.95 [0.92-0.99]  0.89 [0.85-0.93] | 1.05 [1.02-1.08]  0.98 [0.95-1.01] | 1.10-[1.08 [1.13]  1.08 [1.05-1.11] | 0.95 [0.88-1.03]  1.01 [0.93-1.10] | 0.91 [0.88-0.94]  0.87 [0.84-0.90] | 0.99 [0.94-1.04]  0.94 [0.90-0.99] |
| **Caesarean Section**  Crude OR [99% CI]  aOR* [99% CI] | 1.08 [1.03-1.13]  1.10 [1.05-1.15] | 1.06 [1.02-1.11]  1.11 [1.06-1.16] | 0.97 [0.92-1.02]  1.01 [0.95-1.07] | 0.95 [0.92-0.99]  0.96 [0.93-0.998] | 0.94 [0.89-0.99]  0.94 [0.89-0.995] | 0.85 [0.82-0.87]  0.84 [0.81-0.86] | 0.96 [0.93-0.99]  0.91 [0.88-0.94] | 1.06 [1.04-1.09]  1.00 [0.98-1.03] | 1.00 [0.98-1.02]  1.00 [0.98-1.03] | 0.97 [0.91-1.04]  1.01 [0.95-1.08] | 1.05 [1.02-1.08]  1.03 [0.999-1.05] | 1.16 [1.11-1.20]  1.13 [1.09-1.18] |
| **Prelabour CS**  Crude OR [99% CI]  aOR* [99% CI] | 1.01 [0.94-1.09]  1.05 [0.98-1.13] | 1.03 [0.96-1.10]  1.05 [0.98-1.12] | 0.97 [0.90-1.05]  0.98 [0.90-1.06] | 1.00 [0.95-1.05]  0.98 [0.93-1.03] | 0.89 [0.82-0.96]  0.88 [0.81-0.96] | 0.79 [0.75-0.82]  0.76 [0.73-0.79] | 0.99 [0.95-1.04]  0.94 [0.90-0.99] | 1.04 [1.01-1.08]  1.03 [0.99-1.07] | 0.99 [0.96-1.02]  1.03 [0.99-1.07] | 1.01 [0.92-1.11]  1.04 [0.95-1.14] | 1.09 [1.05-1.13]  1.07 [1.03-1.12] | 1.27 [1.20-1.34]  1.28 [1.22-1.35] |
| **Intrapartum CS** *(women without prelabour CS)*  Crude OR [99% CI]  aOR* [99% CI] | 1.12 [1.06-1.19]  1.13 [1.06-1.20] | 1.08 [1.02-1.14]  1.15 [1.08-1.22] | 0.97 [0.90-1.04]  1.03 [0.96-1.11] | 0.93 [0.89-0.97]  0.95 [0.91-1.00] | 0.98 [0.92-1.04]  0.99 [0.92-1.06] | 0.90 [0.86-0.93]  0.90 [0.87-0.94] | 0.93 [0.90-0.97]  0.90 [0.86-0.94] | 1.07 [1.04-1.10]  0.99 [0.96-1.02] | 1.01 [0.98-1.04]  0.98 [0.95-1.01] | 0.95 [0.88-1.03]  1.00 [0.92-1.08] | 1.02 [0.99-1.06]  0.99 [0.96-1.03] | 1.07 [1.02-1.12]  1.03 [0.98-1.08] |
| **Involvement paediatrician <24 hrs**  Crude OR [99% CI]  aOR* [99% CI] | 1.30 [1.25-1.34]  1.30 [1.26-1.35] | 0.92 [0.89-0.95]  0.95 [0.92-0.98] | 0.98 [0.84-1.02]  1.01 [0.97-1.05] | 0.87 [0.84-0.89]  0.88 [0.85-0.90] | 0.88 [0.84-0.91]  0.89 [0.86-0.93] | 1.11 [1.09-1.13]  1.11 [1.09-1.13] | 1.32 [1.29-1.35]  1.28 [1.25-1.31] | 0.59 [0.58-0.60]  0.55 [0.54-0.56] | 1.01 [0.995-1.03]  0.99 [0.97-1.004] | 0.91 [0.87-0.95]  0.93 [0.89-0.98] | 1.06 [1.04-1.08]  1.05 [1.03-1.07] | 1.37 [1.33-1.41]  1.36 [1.32-1.40] |
| ***Women in midwife-led care at onset of labour, n = 328,009*** | | | | | | | | | | | | |
|  | **GR** | **FR** | **DR** | **OV** | **FL** | **GD** | **UT** | **NH** | **ZH** | **ZL** | **NB** | **LB** |
| **Total *n*** | 10,013 | 12,901 | 7,884 | 23,922 | 8,942 | 41,401 | 30,601 | 59,750 | 70,023 | 6,068 | 42,040 | 14,464 |
| **Augmentation of labour after spontaneous onset**  Crude OR [99% CI]  aOR* [99% CI] | 0.98 [0.93-1.04]  0.98 [0.92-1.05] | 0.86 [0.82-0.91]  0.94 [0.89-0.995] | 1.02 [0.95-1.08]  1.11 [1.03-1.19] | 0.78 [0.74-0.81]  0.80 [0.76-0.83] | 1.24 [1.17-1.31]  1.20 [1.13-1.29] | 1.08 [1.05-1.12]  1.12 [1.08-1.16] | 1.19 [1.15-1.23]  1.16 [1.11-1.20] | 1.16 [1.13-1.19]  1.04 [1.003-1.07] | 1.02 [0.99-1.05]  0.94 [0.92-0.97] | 0.68 [0.63-0.74]  0.71 [0.66-0.78] | 1.18 [1.14-1.21]  1.14 [1.12-1.18] | 1.00 [0.95-1.05]  0.99 [0.94-1.04] |
| **Epidural**  Crude OR [99% CI]  aOR* [99% CI] | 1.18 [1.09-1.28]  1.19 [1.09-1.29] | 0.79 [0.73-0.86]  0.87 [0.79-0.95] | 0.41 [0.36-0.47]  0.43 [0.37-0.49] | 1.07 [1.01-1.13]  1.17 [1.10-1.24] | 0.49 [0.44-0.55]  0.43 [0.37-0.49] | 1.21 [1.16-1.27]  1.29 [1.23-1.35] | 1.56 [1.49-1.63]  1.57 [1.49-1.65] | 1.01 [0.97-1.05]  0.85 [0.81-0.89] | 1.07 [1.03-1.11]  0.94 [0.90-0.98] | 0.75 [0.67-0.85]  0.81 [0.72-0.92] | 1.92 [1.84-2.00]  1.97 [1.89-2.06] | 1.68 [1.59-1.79]  1.78 [1.67-1.90] |
| **Other pharmacological pain relief**  Crude OR [99% CI]  aOR* [99% CI] | 0.77 [0.71-0.84]  0.78 [0.72-0.85] | 0.85 [0.79-0.91]  0.91 [0.85-0.98] | 1.68 [1.56-1.80]  1.80 [1.67-1.94] | 0.67 [0.64-0.71]  0.69 [0.65-0.74] | 2.14 [2.01-2.28]  2.03 [1.89-2.17] | 0.76 [0.73-0.80]  0.78 [0.74-0.81] | 0.70 [0.67-0.74]  0.67 [0.63-0.71] | 1.20 [1.16-1.24]  1.13 [1.08-1.17] | 0.98 [0.95-1.01]  0.92 [0.88-0.95] | 0.91 [0.83-1.01]  0.96 [0.86-1.06] | 0.92 [0.88-0.95]  0.90 [0.87-0.94] | 1.21 [1.14-1.28]  1.22 [1.14-1.29] |
| **Instrumental vaginal birth**  Crude OR [99% CI]  aOR* [99% CI] | 1.13 [1.04-1.22]  1.11 [1.02-1.22] | 0.99 [0.92-1.07]  1.06 [0.98-1.15] | 1.01 [0.92-1.11]  1.05 [0.95-1.17] | 1.04 [0.98-1.10]  1.10 [1.03-1.17] | 1.04 [0.95-1.14]  1.09 [0.99-1.21] | 0.93 [0.89-0.98]  0.93 [0.88-0.98] | 0.97 [0.92-1.02]  0.90 [0.85-0.95] | 1.07 [1.03-1.12]  0.99 [0.94-1.03] | 1.04 [0.997-1.08]  1.02 [0.97-1.06] | 0.89 [0.80-1.001]  0.96 [0.86-1.09] | 0.96 [0.92-1.01]  0.90 [0.85-0.94] | 0.96 [0.89-1.04]  0.93 [0.86-1.01] |
| **Intrapartum CS**  Crude OR [99% CI]  aOR* [99% CI] | 1.18 [1.06-1.31]  1.17 [1.05-1.31] | 1.16 [1.05-1.27]  1.26 [1.14-1.39] | 0.85 [0.74-0.97]  0.90 [0.78-1.05] | 0.92 [0.85-0.99]  0.97 [0.89-1.05] | 1.10 [0.97-1.23]  1.07 [0.94-1.23] | 0.89 [0.84-0.95]  0.90 [0.85-0.97] | 0.97 [0.91-1.04]  0.93 [0.86-0.998] | 1.15 [1.09-1.21]  1.03 [0.97-1.09] | 0.99 [0.94-1.04]  0.97 [0.91-1.02] | 0.93 [0.80-1.08]  1.00 [0.86-1.17] | 1.00 [0.94-1.07]  0.95 [0.89-1.01] | 0.95 [0.86-1.05]  0.92 [0.83-1.02] |
| **Involvement paediatrician <24 hrs**  Crude OR [99% CI]  aOR* [99% CI] | 1.26 [1.19-1.33]  1.28 [1.21-1.35] | 0.94 [0.89-0.98]  0.99 [0.94-1.04] | 0.92 [0.86-0.98]  0.95 [0.88-1.01] | 0.83 [0.80-0.86]  0.84 [0.81-0.88] | 0.96 [0.91-1.02]  0.94 [0.88-1.01] | 1.14 [1.11-1.17]  1.16 [1.13-1.20] | 1.43 [1.38-1.48]  1.43 [1.38-1.48] | 0.62 [0.61-0.64]  0.56 [0.55-0.58] | 0.94 [0.92-0.97]  0.91 [0.88-0.93] | 0.75 [0.70-0.81]  0.77 [0.72-0.84] | 1.18 [1.15-1.22]  1.16 [1.12-1.19] | 1.37 [1.31-1.43]  1.39 [1.33-1.46] |
| ***Women in obstetrician-led care at onset of labour*** | | | | | | | | | | | | |
|  | **GR** | **FR** | **DR** | **OV** | **FL** | **GD** | **UT** | **NH** | **ZH** | **ZL** | **NB** | **LB** |
| **Total *n*** | 9,376 | 9,596 | 7,952 | 18,718 | 8,476 | 29,571 | 22,078 | 45,077 | 68,223 | 5,222 | 41,903 | 16,706 |
| **Induction of labour**  Crude OR [99% CI]  aOR* [99% CI] | 1.22 [1.16-1.28]  1.18 [1.12-1.24] | 1.05 [1.002-1.11]  1.00 [0.95-1.05] | 1.27 [1.20-1.34]  1.22 [1.15-1.29] | 1.14 [1.10-1.19]  1.11 [1.07-1.15] | 0.97 [0.92-1.03]  1.03 [0.97-1.09] | 0.93 [0.90-0.96]  0.91 [0.88-0.94] | 0.84 [0.81-0.87]  0.87 [0.84-0.90] | 0.91 [0.88-0.93]  0.99 [0.96-1.02] | 0.97 [0.94-0.99]  1.05 [1.02-1.07] | 1.05 [0.98-1.12]  1.00 [0.93-1.07] | 0.86 [0.84-0.88]  0.87 [0.84-0.89] | 0.90 [0.87-0.94]  0.87 [0.83-0.90] |
| **Augmentation of labour after spontaneous onset**  Crude OR [99% CI]  aOR* [99% CI] | 0.87 [0.80-0.95]  0.89 [0.81-0.97] | 0.94 [0.87-1.02]  1.01 [0.92-1.10] | 1.02 [0.93-1.11]  1.05 [0.95-1.16] | 0.87 [0.82-0.93]  0.88 [0.82-0.94] | 1.41 [1.30-1.53]  1.43 [1.30-1.56] | 1.06 [1.01-1.11]  1.09 [1.03-1.14] | 0.95 [0.90-0.998]  0.93 [0.88-0.99] | 1.11 [1.06-1.15]  1.05 [1.01-1.10] | 1.09 [1.05-1.13]  1.03 [0.99-1.08] | 0.77 [0.69-0.86]  0.79 [0.70-0.88] | 1.08 [1.04-1.13]  1.07 [1.03-1.12] | 0.97 [0.91-1.03]  0.92 [0.86-0.98] |
| **Intrapartum oxytocin use**  Crude OR [99% CI]  aOR* [99% CI] | 1.13 [1.07-1.18]  1.12 [1.07-1.18] | 1.09 [1.04-1.15]  1.11 [1.05-1.17] | 1.28 [1.21-1.35]  1.29 [1.22-1.37] | 0.95 [0.92-0.99]  0.94 [0.91-0.98] | 1.31 [1.24-1.38]  1.34 [1.26-1.42] | 1.02 [0.99-1.06]  1.03 [0.99-1.06] | 0.95 [0.91-0.98]  0.96 [0.93-0.996] | 0.97 [0.95-0.999]  0.99 [0.97-1.02] | 0.96 [0.94-0.98]  0.97 [0.94-0.99] | 0.82 [0.76-0.87]  0.80 [0.75-0.85] | 0.82 [0.80-0.84]  0.82 [0.79-0.84] | 0.85 [0.81-0.88]  0.81 [0.78-0.84] |
| **Epidural** *(women without prelabour CS)*  Crude OR [99% CI]  aOR* [99% CI] | 1.01 [0.95-1.07]  1.01 [0.94-1.08] | 0.84 [0.79-0.90]  0.88 [0.82-0.94] | 0.48 [0.44-0.52]  0.49 [0.45-0.53] | 1.16 [1.11-1.21]  1.20 [1.15-1.26] | 0.51 [0.47-0.55]  0.48 [0.44-0.52] | 1.22 [1.18-1.26]  1.25 [1.20-1.30] | 1.32 [1.27-1.38]  1.35 [1.29-1.41] | 0.97 [0.94-1.002]  0.92 [0.89-0.95] | 1.23 [1.19-1.26]  1.14 [1.11-1.18] | 0.82 [0.75-0.90]  0.84 [0.77-0.92] | 1.63 [1.58-1.68]  1.67 [1.61-1.72] | 1.64 [1.57-1.71]  1.63 [1.56-1.70] |
| **Other pharmacological pain relief** *(women without prelabour CS)*  Crude OR [99% CI]  aOR* [99% CI] | 0.83 [0.78-0.89]  0.82 [0.77-0.87] | 0.98 [0.92-1.04]  0.99 [0.93-1.05] | 1.87 [1.77-1.98]  1.86 [1.75-1.97] | 0.71 [0.68-0.75]  0.71 [0.68-0.75] | 2.28 [2.16-2.41]  2.23 [2.11-2.37] | 0.72 [0.70-0.75]  0.73 [0.71-0.76] | 0.56 [0.53-0.58]  0.55 [0.53-0.58] | 0.97 [0.94-1.00]  1.01 [0.97-1.04] | 0.98 [0.95-1.001]  0.98 [0.95-1.01] | 1.14 [1.06-1.23]  1.13 [1.05-1.22] | 0.73 [0.70-0.75]  0.73 [0.71-0.76] | 1.27 [1.22-1.33]  1.26 [1.20-1.31] |
| **Instrumental vaginal birth** *(women without prelabour CS)*  Crude OR [99% CI]  aOR* [99% CI] | 1.09 [1.001-1.18]  1.10 [1.01-1.19] | 1.05 [0.97-1.14]  1.10 [1.01-1.21] | 0.91 [0.82-0.99]  0.92 [0.83-1.01] | 1.17 [1.10-1.24]  1.21 [1.13-1.28] | 0.88 [0.80-0.97]  0.95 [0.86-1.05] | 0.96 [0.92-1.02]  0.97 [0.92-1.02] | 0.97 [0.92-1.03]  0.91 [0.86-0.98] | 1.05 [1.004-1.10]  1.00 [0.95-1.05] | 1.15 [1.11-1.19]  1.12 [1.08-1.17] | 1.01 [0.90-1.13]  1.05 [0.93-1.18] | 0.85 [0.81-0.89]  0.83 [0.79-0.87] | 0.98 [0.92-1.04]  0.92 [0.86-0.98] |
| **Prelabour CS**  Crude OR [99% CI]  aOR* [99% CI] | 0.96 [0.90-1.04]  1.01 [0.93-1.09] | 1.14 [1.06-1.22]  1.14 [1.06-1.23] | 0.88 [0.81-0.96]  0.87 [0.80-0.95] | 1.06 [1.003-1.12]  1.04 [0.99-1.10] | 0.84 [0.77-0.91]  0.82 [0.75-0.90] | 0.87 [0.83-0.91]  0.84 [0.80-0.88] | 1.12 [1.07-1.18]  1.07 [1.02-1.13] | 1.15 [1.11-1.19]  1.15 [1.11-1.20] | 0.93 [0.90-0.96]  0.98 [0.94-1.02] | 1.02 [0.93-1.12]  1.04 [0.94-1.14] | 1.02 [0.98-1.06]  1.00 [0.96-1.04] | 1.09 [1.03-1.15]  1.11 [1.05-1.18] |
| **Intrapartum CS** *(women without prelabour CS)*  Crude OR [99% CI]  aOR* [99% CI] | 1.06 [0.99-1.14]  1.07 [0.996-1.16] | 1.15 [1.07-1.23]  1.20 [1.12-1.30] | 0.94 [0.87-1.02]  0.99 [0.91-1.07] | 0.99 [0.94-1.04]  1.00 [0.94-1.06] | 0.88 [0.81-0.95]  0.90 [0.83-0.98] | 0.97 [0.93-1.02]  0.98 [0.93-1.02] | 1.01 [0.96-1.06]  0.98 [0.93-1.04] | 1.13 [1.09-1.17]  1.07 [1.03-1.11] | 0.97 [0.94-1.00]  0.95 [0.91-0.98] | 0.97 [0.88-1.07]  0.99 [0.90-1.10] | 0.98 [0.94-1.02]  0.96 [0.92-0.998] | 0.99 [0.94-1.05]  0.95 [0.89-1.003] |
| **Involvement paediatrician <24 hours**  Crude OR [99% CI]  aOR* [99% CI] | 1.36 [1.28-1.43]  1.36 [1.28-1.44] | 0.99 [0.94-1.05]  1.00 [0.95-1.06] | 0.91 [0.86-0.96]  0.92 [0.86-0.97] | 0.94 [0.90-0.97]  0.94 [0.91-0.98] | 0.72 [0.69-0.76]  0.75 [0.71-0.80] | 1.34 [1.30-1.39]  1.34 [1.29-1.38] | 1.58 [1.52-1.64]  1.56 [1.49-1.62] | 0.54 [0.53-0.55]  0.53 [0.51-0.54] | 0.98 [0.96-1.001]  0.97 [0.94-0.999] | 1.04 [0.97-1.12]  1.05 [0.97-1.12] | 0.88 [0.85-0.90]  0.87 [0.85-0.90] | 1.19 [1.14-1.24]  1.17 [1.12-1.22] |

*Odds ratios, adjusted for parity, maternal age, ethnic background, socioeconomic status and urbanisation
